# Supplementary material for: A phased small interfering RNA acts in an OsAGO2-dependent manner to mediate OsARF7 cleavage and promote male fertility in rice
Source: Plant Commun. 2026 Mar 3;7(8):101788. doi: 10.1016/j.xplc.2026.101788 (PMC13477049; doi:10.1016/j.xplc.2026.101788)
Supplement: Supplementary file 1 [file mmc1.pdf]

Plant Communications, Volume *7*

Supplemental information

A phased small interfering RNA acts in an OsAGO2-dependent manner to mediate *OsARF7* cleavage and promote male fertility in rice

Liang Shao, Keli Qiu, Rui Xu, Jiankai Zhou, Junwen Yang, Jun Liu, Yunfei Yang, Lingling Li, Biao Xie, Yu Wang, Jiaojiao Li, Xiaoshuang Liu, Meng Yuan, Yachun Yang, Yunfei Li, Pengcheng Wei, Haiyang Jiang, Yijun Qi, and Pengfei Jiang

A phased small interfering RNA acts in an OsAGO2-dependent manner to mediate *OsARF7* cleavage and promote male fertility in rice

Liang Shao[1,6], Keli Qiu[1,6], Rui Xu[1,6], Jiankai Zhou[1,6], Junwen Yang[1], Jun Liu[1], Yunfei Yang[1], Lingling Li[1], Biao Xie[1], Yu Wang[1,3], Jiaojiao Li[4], Xiaoshuang Liu[5], Meng Yuan[2], Yachun Yang[3], Yunfei Li[1], Pengcheng Wei[1,5]*, Haiyang Jiang[1]*, Yijun Qi[2]*, Pengfei Jiang[1]*

1. National Engineering Laboratory of Crop Stress Resistance Breeding, School of Life Sciences, Anhui Agricultural University, Hefei 230036, China

2. Center for Plant Biology, School of Life Sciences, Tsinghua University, Beijing 100084, China.

3. Anhui Province Key Laboratory of Rice Germplasm Innovation and Molecular Improvement, Anhui Academy of Agricultural Sciences, Hefei 230036, China

4. CAS Center for Excellence in Molecular Plant Sciences, School of Life Sciences, University of Science and Technology of China, Huangshan Road 443, Hefei 230027, China

5. College of Agronomy, Anhui Agricultural University, Hefei 230036, China

6. These authors contribute equally to this work.

*Correspondence: Pengcheng Wei (weipengcheng@gmail.com), Haiyang Jiang (hyjiang@ahau.edu.cn), Yijun Qi (qiyijun@tsinghua.edu.cn), Pengfei Jiang (jiangpengfei@ahau.edu.cn)

Figure S1. Seed-setting rate and vegetative growth of *osphas1-1* and *osphasiR1_1-STTM* mutants

Figure S2. Phylogenetic analysis of *OsARFs* and sequence complementarity with osphasiR1_1

Figure S3. Seed setting rate of *OsARF7^{WT}-OE* and *OsARF7^{mu}-OE* lines

Figure S4. Loss of function of *OsARF7* results in normal male fertility

Figure S5. PCR-based genome sequencing of *osago2* and *osago5c* at Cas9 target region

Figure S6. The male fertility and *OsARF7* expression in *osago2* and *osago5c* mutants

Figure S7. Seed setting rate of *osago2-3* and *osago2-3 OsARF7-RNAi* lines

Figure S8. Heat map of the top 100 differentially expressed genes (DEGs)

Figure S9. Expression of fertility-related genes in WT and *OsARF7^{mu}*-OE lines

Figure S10. Expression of *OsARF7* during anther development in *osphas1-1* and *OsARF7^{mu}-OE*#3

Supplementary Table 1. Information of phasiRNAs which have targets degradome signal at top 30 in different rice varieties

Supplementary Table 2. Primers used in this study

Supplementary Table 3. Summary of the RNA-Seq data

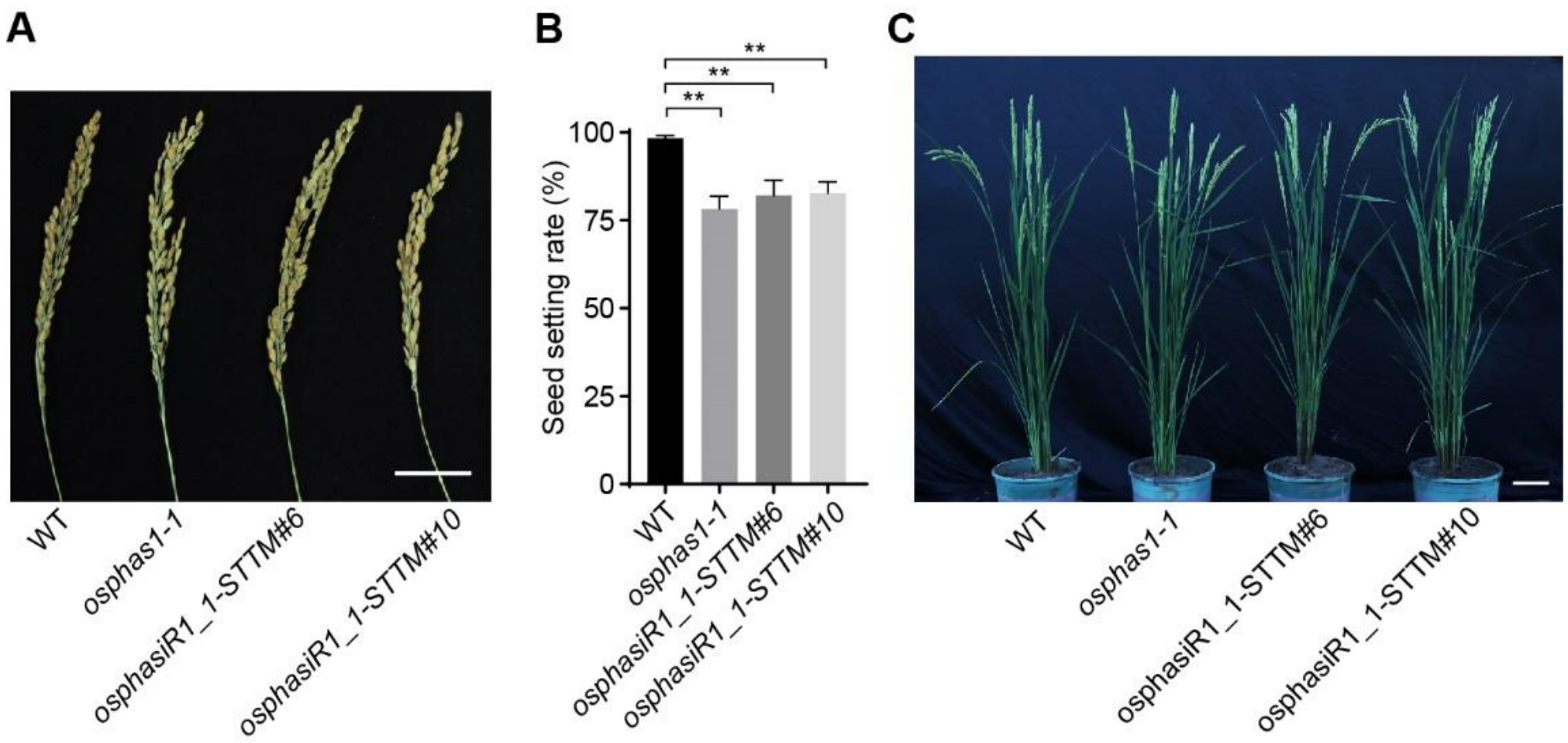

Figure S1. Seed-setting rate and vegetative growth of *osphas1-1* and *osphasiR1_1-STTM* mutants

(A-B) Panicles and Statistical analysis of seed-setting rate of WT and osphasiR1_1 mutants. Each two panicles from 5 seedlings of each mutant were used, t-test **P < 0.01. Bar = 4 cm.

(C) Representative images of 83-day-old WT, *osphas1-1*, and osphasiR1_1-STTM lines grown under long-day conditions (Hefei, China). Bar = 10 cm.

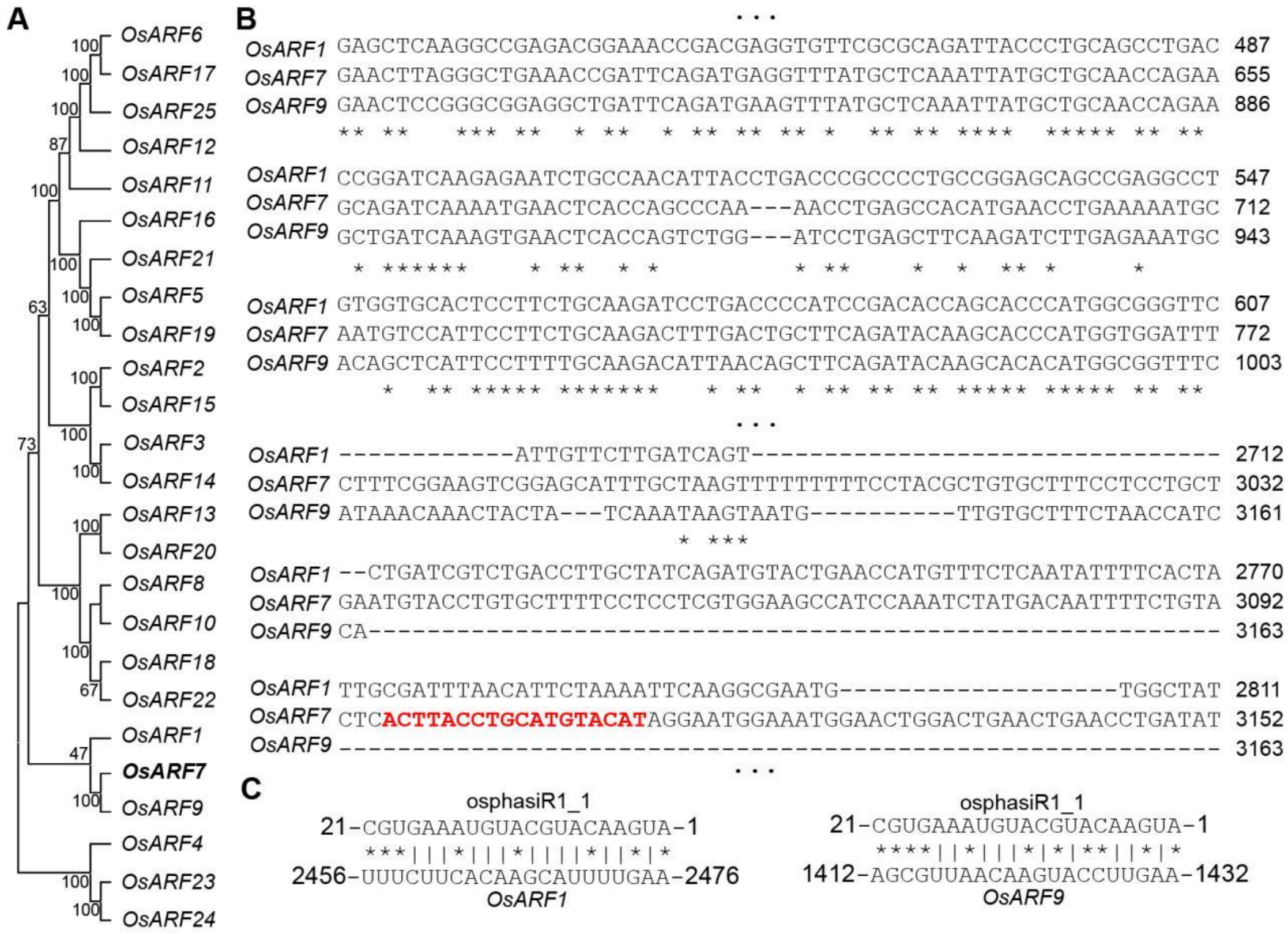

Figure S2. Phylogenetic analysis of *OsARFs* and sequence complementarity with osphasiR1_1

(A) Phylogenetic tree of *OsARFs* constructed using Neighbor-Joining (NJ) method.

(B) Sequence alignment of *OsARF1*, *OsARF7* and *OsARF9*. The sequences of osphasiR1_1 are shown in red.

(C) The mRNA sequences of *OsARF1* and *OsARF9* that exhibit maximum complementarity to osphasiR1_1.

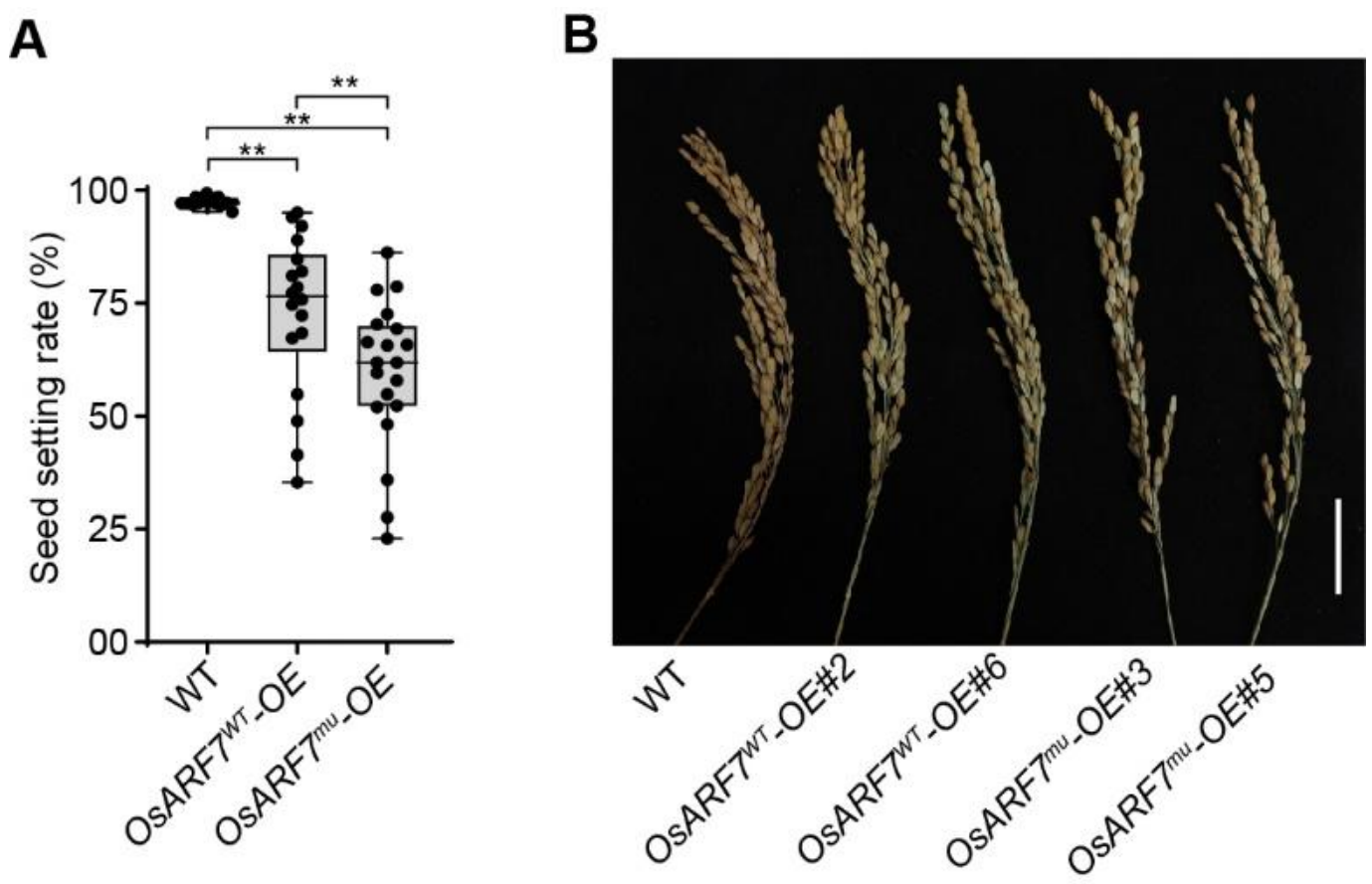

Figure S3. Seed setting rate of *OsARF7^{WT}-OE* and *OsARF7^{mu}-OE* lines

(A) Seed setting rate of WT, *OsARF7^{WT}-OE* and *OsARF7^{mu}-OE* lines at T_2 generation. At least 3 homozygous plants of each T_2 line were used, and each black solid dot indicates the average value of one line. T-test, $^{**}P < 0.01$.

(B) Panicles of WT as well as each two *OsARF7^{WT}-OE* and *OsARF7^{mu}-OE* lines after grain filling. Bar = 4 cm.

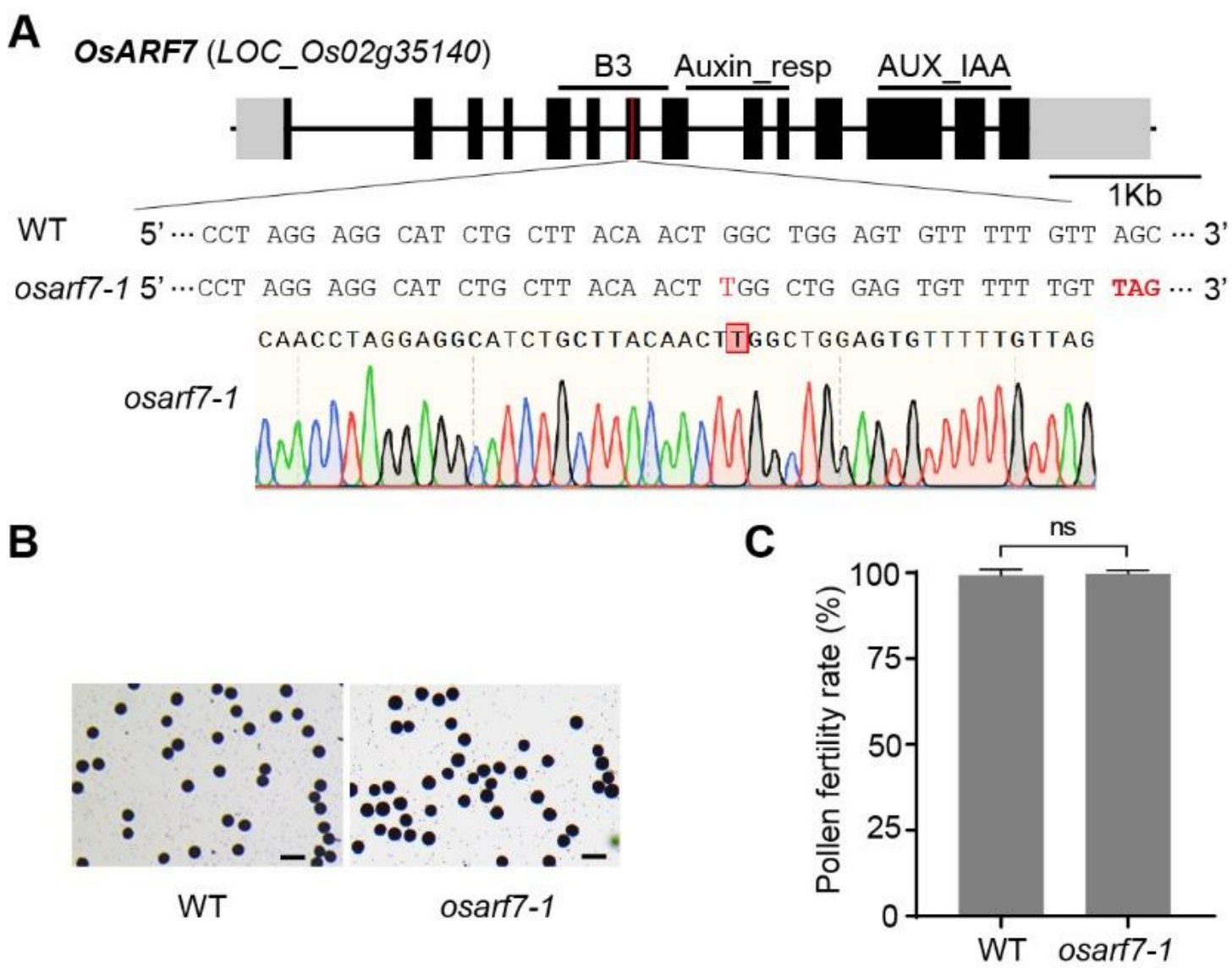

Figure S4. Loss of function of *OsARF7* results in normal male fertility

(A) Gene structure of *OsARF7*. The red vertical lines indicate the Cas9 target site of *OsARF7*.

(B-C) I_2-KI staining and statistics of pollen fertility of WT and *osarf7-1* mutant. Ten plants and 10 anthers per plant were used. T-test, ns indicates no significant difference. Bar = 200 μm

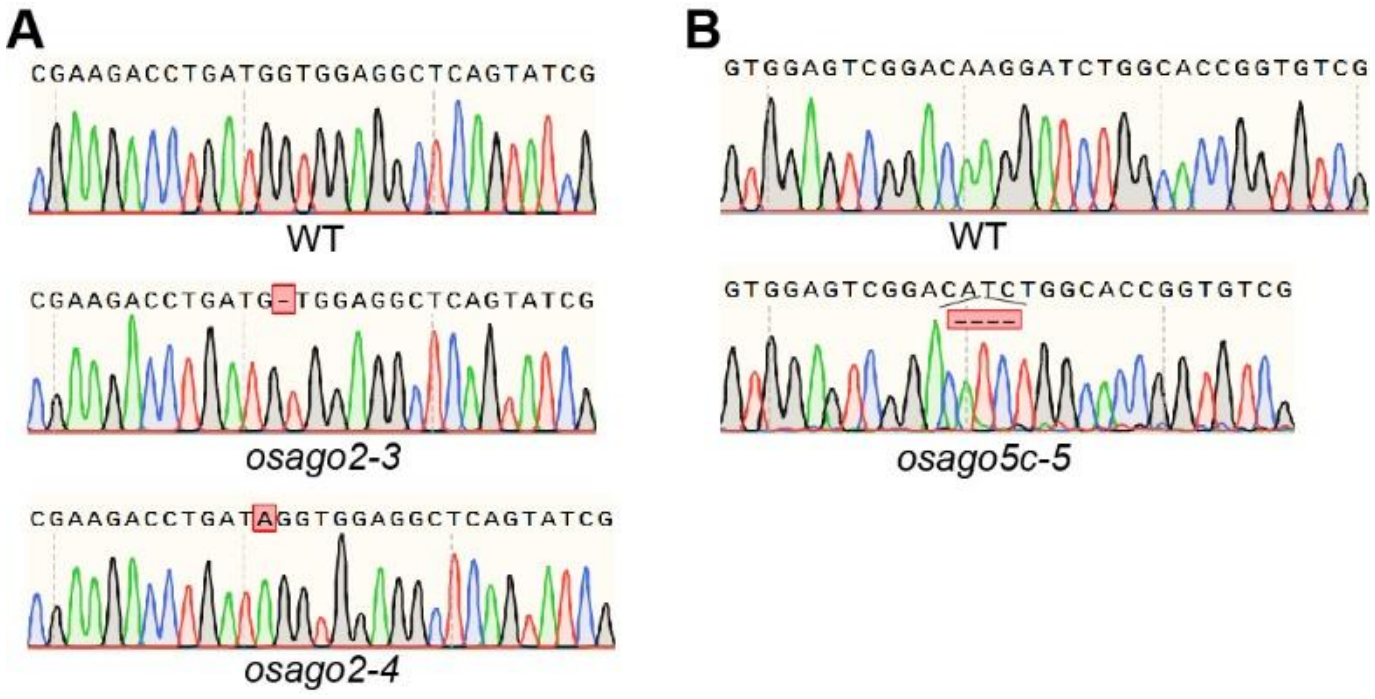

Figure S5. PCR-based genome sequencing of *osago2* and *osago5c* at Cas9 target region

Black lines highlighted in red background indicate deleted bases in the mutants, and nucleotide highlighted in red background indicates inserted base in the mutants.

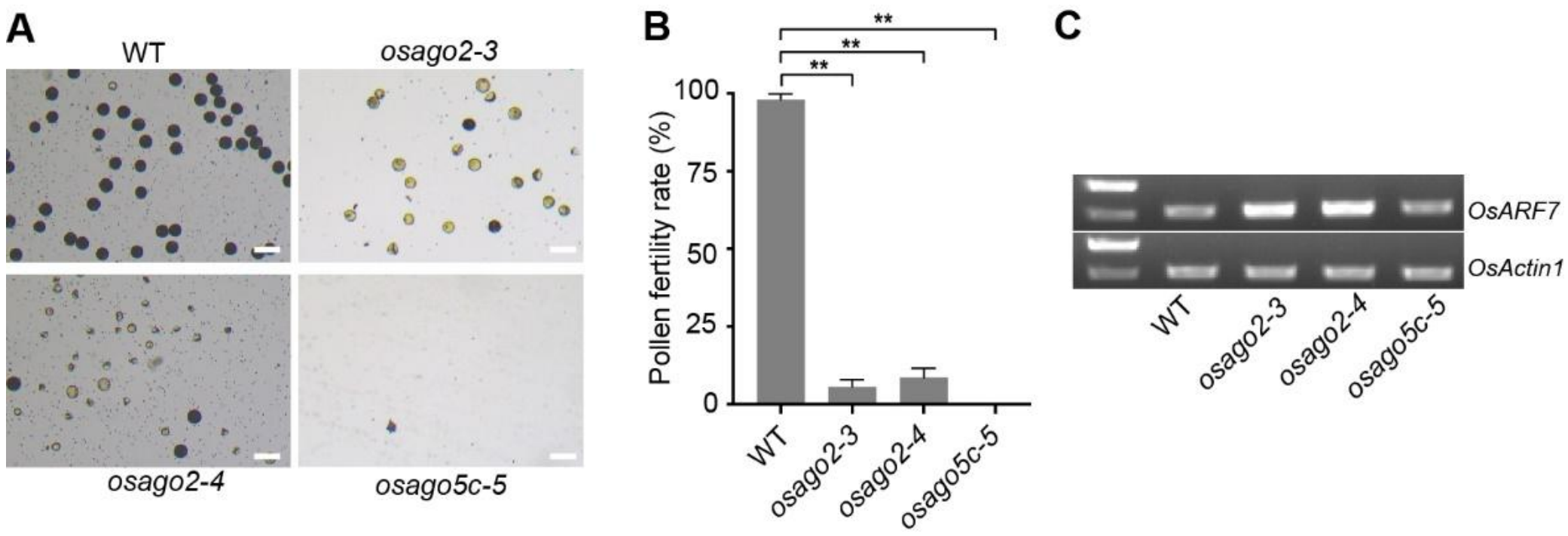

Figure S6. The male fertility and *OsARF7* expression in *osago2* and *osago5c* mutants

(A-B) I_2-KI staining and statistics of pollen fertility of WT, *osago2* and *osago5c* mutants. Ten plants and 10 anthers per plant were used. T-test, $^{**}P < 0.01$. Bar = 200 µm.

(C) Semi-quantitative PCR analysis of *OsARF7* expression in *osago2* and *osago5c* mutants.

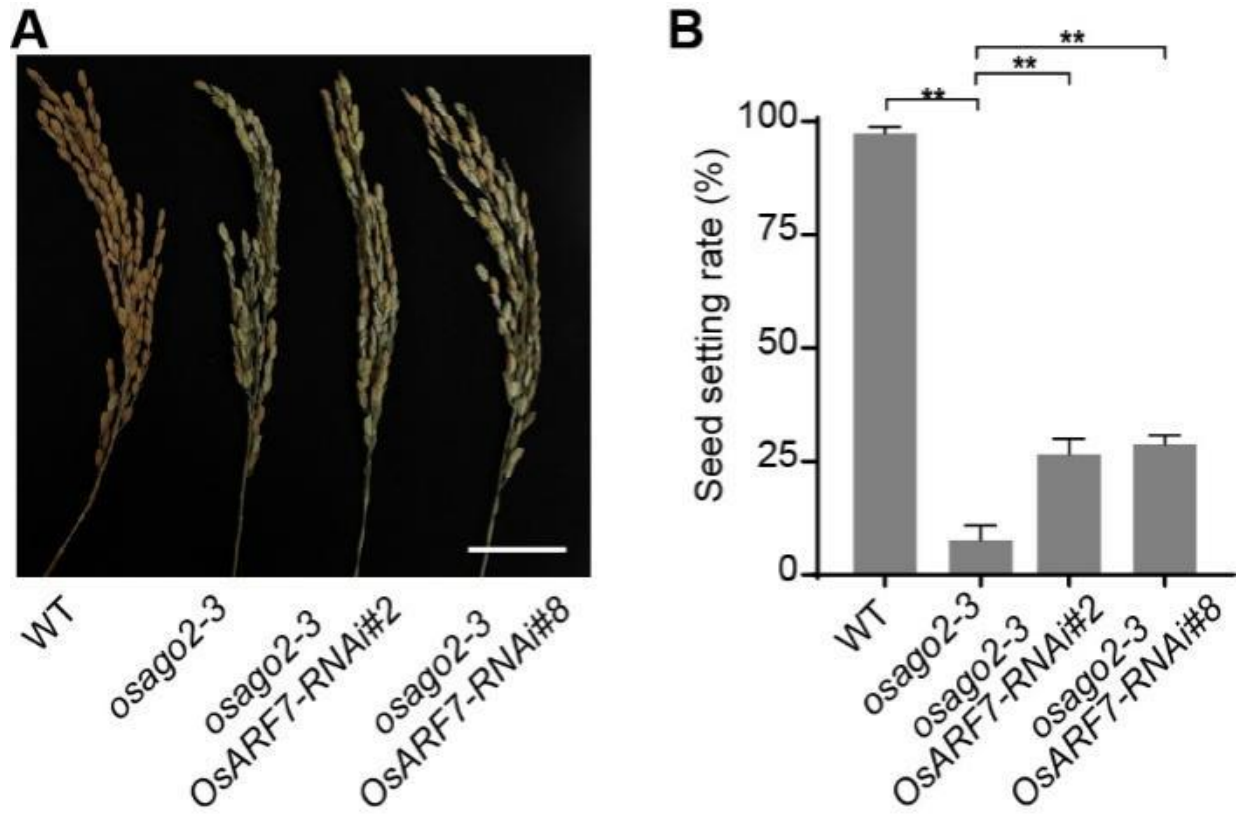

Figure S7. Seed setting rate of *osago2-3* and *osago2-3 OsARF7-RNAi* lines

(A) Panicles of WT, *osago2-3* and *osago2-3 OsARF7-RNAi* lines after grain filling. Bar = 4 cm.

(B) Seed setting rate. Ten plants and 3 panicles per plant were used. T-test, **P < 0.01.

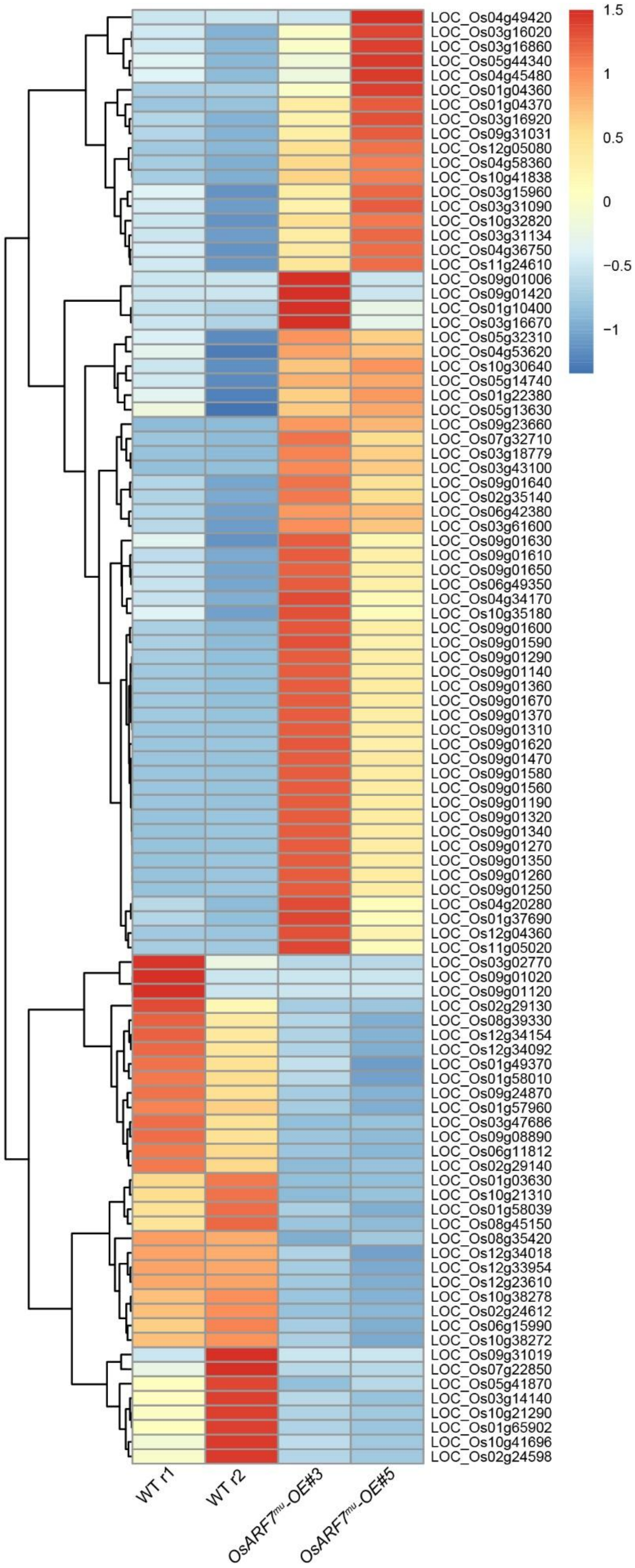

Figure S8. Heat map of the top 100 differentially expressed genes (DEGs)

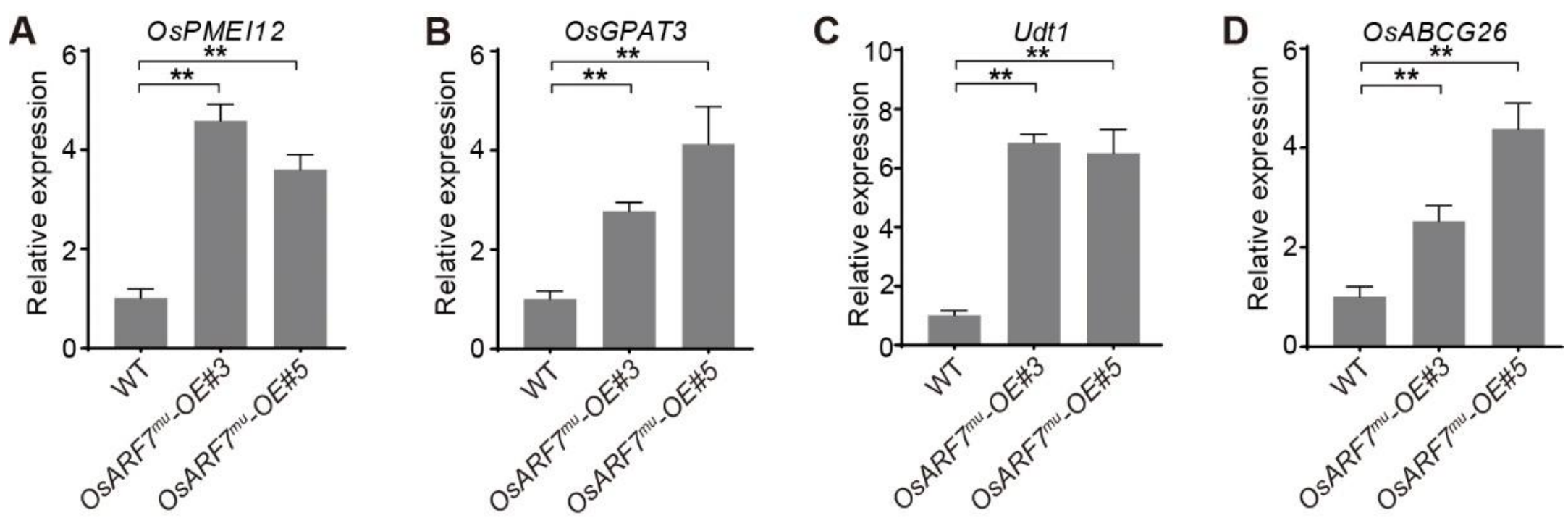

Figure S9. Expression of fertility-related genes in WT and *OsARF7^{mu}-OE* lines

N=3, biological replicates, t-test, **P < 0.01.

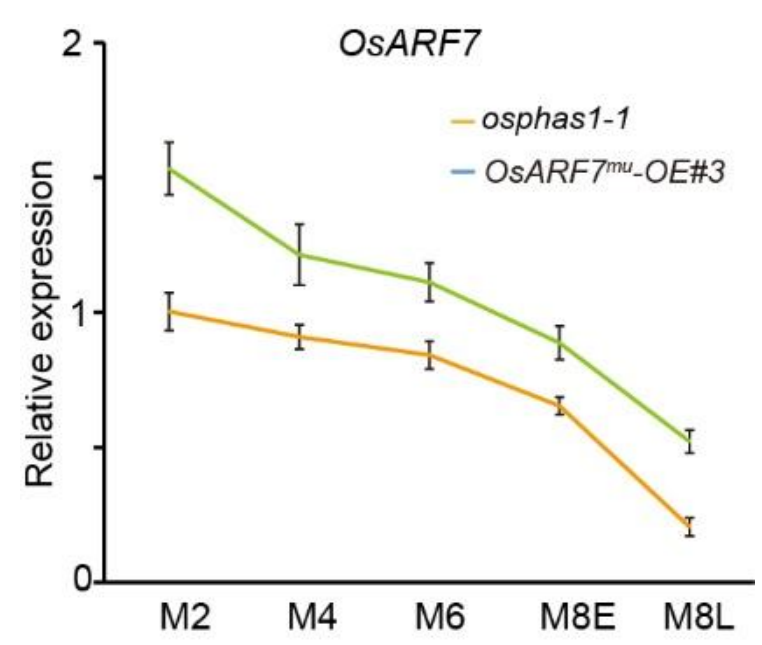

Figure S10. Expression of *OsARF7* during anther development in *osphas1-1* and *OsARF7^{mu}-OE*#3

N=3, biological replicates.

Supplementary Table 1. Information of phasiRNAs which have targets degradome signal at top 30 in different rice varieties

*phasiRNA*_ID	5' base	Target_ID	Cleavage site	Nipponbare		3037		HHZ		target description
				Total counts	Ranking	Total counts	Ranking	Total counts	Ranking	
21PHAS282_404(+)	*C*	LOC_Os12g33962	1031	6984	**1st**	3365	**1st**	2436	**1st**	tatC-like protein ymf16,
21PHAS672_488(-)	C	LOC_Os03g18820	1832	1390	**3rd**	2090	**2nd**	1787	**2nd**	glycosyltransferase
21PHAS277_639(+)	C	LOC_Os12g42550	1159	537	**9th**	1799	**3rd**	1053	**3rd**	methyl-CpG binding
21PHAS16_214(-)	C	LOC_Os02g02870	1193	732	**6th**	1197	**4th**	885	**4th**	glycine-rich protein 2
21PHAS511_132(-)	C	LOC_Os03g48930	776	1496	**2nd**	684	**5th**	673	**5th**	peptidase, T1 family
21PHAS1035_156(-)	*C*	LOC_Os06g44374	341	417	**13th**	283	**17th**	524	**6th**	SEC61 subunit gamma
21PHAS466_247(-) /osphasiR1_1	A	**LOC_Os02g35140**	**3104**	**486**	**10th**	**555**	**8th**	**480**	**7th**	**auxin response factor 7**
21PHAS46_349(-)	C	LOC_Os04g58890	1372	839	**5th**	581	**7th**	427	**8th**	expressed protein
21PHAS108_172(-)	G	LOC_Os10g02980	1574	262	**17th**	408	**10th**	379	**10th**	formin, putative
21PHAS1643_235(-)	C	LOC_Os05g39090	1994	290	**15th**	460	**9th**	340	**11th**	CAMK_CAMK_like.29
21PHAS1826_79(-)	C	LOC_Os02g47410	3529	691	**7th**	404	**11th**	211	**14th**	protein kinase
21PHAS944_81(+)	*C*	LOC_Os09g31970	1908	269	**16th**	290	**16th**	150	**19th**	HMGR putative

Supplementary Table 2. Primers used in this study

Gene	Primer	Sequence
Genotyping		
pUBI	F-primer	AATGCCAGCCTGTTAAACGCCGT
pUBI	R-primer	TGACCCGACAAACAAGTGCACG
osphas1-casdele	F-primer	GCATAACCTCATTGACCGAAAG
osphas1-casdele	R-primer	GTAGGACTATTCCCGACACCAC
ago2	F-primer	GCAACATTTGCTCTGTCATTTCC
ago2	R-primer	TTGACACGAAACAAACCATCTG
ago5c	F-primer	GCAGAAGCTGTTCGTGTCGGAGAC
ago5c	R-primer	GGAAAGAGGGTCAGGGGTGAGCT
osarf7	F-primer	GCAAGAGCTTGTGGCTAGAGATCTCC
osarf7	R-primer	GATGAATGCGTCACCGGCAACT
Vector construction		
OsARF7^{WT}-OE	F-primer	CAGCTATGACATGATTACGAATTCCAATGATAGCCAACCAC
OsARF7^{WT}-OE	R-primer	CTGCAGGTCGACTCTAGAGGATCCAGCAAACACGTTTGGAGGTCACG
OsARF7^{mu}-OE	R-primer	TCCTATCTACAAGGTGGTAAGTGAGTACAGAAAATTGTC
OsARF7^{mu}-OE	F-primer	TTACCACCTTGTAGATAGGAATGGAAATGGAACTGGAC
OsphasiR1_1-STTM	F-primer	CTCGGTACCGCACTTTACATCTAGCATGTTCATGTTGTTGTTGTTATGGTCTAGTTGTT
OsphasiR1_1-STTM	R-primer	TCACTGCAGATGAACATGCCTAATGTAAAGTGCATTCTTCTTCTTTAGACCATATTCTT
OsARF7-RNAi	F-primer	GTGCCAAGCTCCTGCAGGCTCGAGGTGGAAATCTTCAGCTGAGTCC
OsARF7-RNAi	R-primer	AGTAGTACGGTCCGTACCAGATCTACACCAGAAACAGCAGCCAGT
BD-ARF7	F-primer	ATATGGCCATGGAGGCCGAATTCATGGCTGGCTCCGTCGTTGCTGCT
BD-ARF7	R-primer	TTATGCGGCCGCTGCAGGTCGACTCAGCAATCCTTATCGGTGACGG
BD-ARF7-N	R-primer	TTATGCGGCCGCTGCAGGTCGACCTAGGACTCCAAGATGCATACTAT

BD-ARF7-C	F-primer	ATATGGCCATGGAGGCCGAATTCTGCATCTTGGAGTCCTAGCAAC
35S-BD-OsARF7	F-primer	CGGCAGGATCCACTAGTGAATTCATGGCTGGCTCCGTCGTTGCTGCT
35S-BD-OsARF7	R-primer	CCGGCCTCGAGGTCGACAAGCTTTCAGCAATCCTTATCGGTGACGG
35S-OsARF7	F-primer	GCCGCTCTAGAACTAGTGGATCCATGGCTGGCTCCGTCGTTGCT
35S-OsARF7	R-primer	AGGTCGACGGTATCGATAAGCTTTCAGCAATCCTTATCGGTGACG
pcHSP70-4	F1-primer	AGGTCGACGGTATCGATAAGCTTAAAACCGCCCTTCATACCACCGAG
pcHSP70-4	R1-primer	GCCGCTCTAGAACTAGTGGATCCCCCTGCCGTCCAAGTGGAGG
pcHSP70-4	F2-primer	AGGTCGACGGTATCGATAAGCTTCCTAAGTGCAAAATGCGCACACCT
pcHSP70-4	R2-primer	GCCGCTCTAGAACTAGTGGATCCCTTTCTTCTCTTCCGATCAAATC
$pUb_{L40}4$	F1-primer	AGGTCGACGGTATCGATAAGCTTATTACAGAGCAAAAAAGGAGT
$pUb_{L40}4$	R1-primer	GCCGCTCTAGAACTAGTGGATCCCGTTTGAAATGTAGATCTGAG
$pUb_{L40}4$	F2-primer	AGGTCGACGGTATCGATAAGCTTTACAAAATATAAAACACATT
$pUb_{L40}4$	R2-primer	GCCGCTCTAGAACTAGTGGATCCCTTGCCGCCGTCGAACGATCT
Stem-loop RT-qPCR		
OsphasiR1_1	RT-primer	GTCGTATCCAGTGCAGGGTCCGAGGTATTCGCACTGGATACGACGCACTT
OsphasiR1_1	F-primer	CGCCGGCATGAACATGCATGTA
OsphasiR1_1	R-primer	CCAGTGCAGGGTCCGAGGT
RT-qPCR		
OsARF7	F-primer	CGCATTCATCTTTCTGAGAGGTG
OsARF7	R-primer	ACTCAGACTGACTTGTTCTTGGT
OsAGO2	F-primer	TGGAGTTGGTGAATGCTGAC
OsAGO2	R-primer	CCACTGGCAGTTAGGCTGATC
cHSP70-4	F-primer	CTTTGATGCAAAGCGGCTGATCG
cHSP70-4	R-primer	ACTTCTTCTCCTCGCCCTTGTACG
OsABCG26	F-primer	TGGCCTCTGAAGCTTGTCAGTTC
OsABCG26	R-primer	GAGGAAATGATCCGATGGATTGCG

Ub$_{L40}$4	F-primer	TTCCCATCAGGTCTCACCACTG
Ub$_{L40}$4	R-primer	TTCTTCTTCAACCTCAGCTCCTTG
OsPMEI12	F-primer	CAAATTCTGCCGGTGCCTTTGC
OsPMEI12	R-primer	TGGCTTTCAATCGCTTGTGGAG
OsGPAT3	F-primer	GTTGGTTGATGTTGTGGGCTTCTC
OsGPAT3	R-primer	TGTCACCAGGTACACCTCCTTG
Udt1	F-primer	CTCTGCAGCTACAAAGTGCAAGTG
Udt1	R-primer	AACATCCTGCTCACCCTTCACCTC
OsActin1	F-primer	ACCCAAGAATGCTAAGCCAAGAG
OsActin1	R-primer	ACTTTGTCCACGCTAATGAAGAAAC
Semi-qRT-PCR		
OsARF7	F-primer	GCTCCAGAACTACCTCCGGTCT
OsARF7	R-primer	TCCTTGCATGATGACCTTGGT
OsAGO2	F-primer	TCGGGTACCGTTGAGCGCATTG
OsAGO2	R-primer	CTTGAAGAGCTCGTCCTTGACAGT
OsActin1	F-primer	AAGTACCCCATCGAGCATGGTATCG
OsActin1	R-primer	GAGCTGGTCTTGGCAGTCTCCATTT
5'RACE		
	5’ RNA adaptor	UUUCCCUACACGACGCUCUUCCGAUCUCCGAC
	RT-dT$_{25}$vn	CGAGCACAGAATTAATACGACT25VN
OsARF7	5'RACE-F1	TTCCCTACACGACGCTCTTC
OsARF7	5'RACE-R1	CGAGCACAGAATTAATACGACT
OsARF7	5'RACE-F2	TACACGACGCTCTTCCGATCT
OsARF7	5'RACE-R2	CACGCAGATTACCCCCATCACG
In stitu hybridization		
OsARF7	F-primer	TGTACAGGGAGCTATGGCATGCTTG
OsARF7	R-primer	GTAATACGACTCACTATAGGGCTGTAGCTTCCACAGCAGAGCTTATC
OsARF7	F- control	GTAATACGACTCACTATAGGGCTGTACAGGGAGCTATGGCATGCTTG
OsARF7	R-control	TGTAGCTTCCACAGCAGAGCTTATC
osphasiR1_1	ISH	5DiGN/GCACTTTACATGCATGTTCAT/3DiGN
osphasiR1_1	ISH-control	5DiGN/ ATGAACATGCATGTAAAGTGC/3DiGN

Supplementary Table 3. Summary of the RNA-Seq data

	**WT r1**	**WT r2**	***OsARF7^{mu}-OE*#3**	***OsARF7^{mu}-OE*#5**
Raw reads	45575108	48041304	22569594	52520112
Q20	97.75	97.59	98.12	98.07
Q30	92.42	91.51	94.39	94.25
Mapped reads	40672347(89.24%)	41311816(85.99%)	20585853(91.21%)	47322364(90.10%)
