# Supplementary material for: A phased small interfering RNA acts in an OsAGO2-dependent manner to mediate OsARF7 cleavage and promote male fertility in rice
Source: Plant Commun. 2026 Mar 3;7(8):101788. doi: 10.1016/j.xplc.2026.101788 (PMC13477049; doi:10.1016/j.xplc.2026.101788)
Supplement: Supplementary file 2 [file mmc2.pdf]

Plant Communications
Research article

CellPress Partner Journal

A phased small interfering RNA acts in an OsAGO2-dependent manner to mediate *OsARF7* cleavage and promote male fertility in rice

Liang Shao[1,6], Keli Qiu[1,6], Rui Xu[1,6], Jiankai Zhou[1,6], Junwen Yang[1], Jun Liu[1], Yunfei Yang[1], Lingling Li[1], Biao Xie[1], Yu Wang[1,3], Jiaojiao Li[4], Xiaoshuang Liu[5], Meng Yuan[2], Yachun Yang[3], Yunfei Li[1], Pengcheng Wei[1,5,*], Haiyang Jiang[1,*], Yijun Qi[2,*] and Pengfei Jiang[1,*]

[1]National Engineering Laboratory of Crop Stress Resistance Breeding, School of Life Sciences, Anhui Agricultural University, Hefei 230036, China

[2]Center for Plant Biology, School of Life Sciences, Tsinghua University, Beijing 100084, China

[3]Anhui Province Key Laboratory of Rice Germplasm Innovation and Molecular Improvement, Anhui Academy of Agricultural Sciences, Hefei 230036, China

[4]CAS Center for Excellence in Molecular Plant Sciences, School of Life Sciences, University of Science and Technology of China, Huangshan Road 443, Hefei 230027, China

[5]College of Agronomy, Anhui Agricultural University, Hefei 230036, China

[6]These authors contributed equally to this article.

***Correspondence: Pengcheng Wei (weipengcheng@gmail.com), Haiyang Jiang (hyjiang@ahau.edu.cn), Yijun Qi (qiyijun@tsinghua.edu.cn), Pengfei Jiang (jiangpengfei@ahau.edu.cn)**

https://doi.org/10.1016/j.xplc.2026.101788

ABSTRACT

A class of small RNAs, the phased small interfering RNAs (phasiRNAs), are specifically expressed in anthers and closely associated with male fertility in monocots. However, the specific 21-nt phasiRNAs involved in regulating male fertility remain unclear. Here, we identified a functional 5′-A phasiRNA (referred to as os-phasiR1_1) that mediates the cleavage of *OsARF7* transcripts to promote male fertility. CRISPR–Cas9 knockout and short tandem target-mimic knockdown of osphasiR1_1 led to significantly reduced male fertility and seed setting. Further mechanistic experiments revealed that osphasiR1_1 mediates *OsARF7* cleavage and promotes male fertility in an OsAGO2-dependent but MEL1/OsAGO5c-independent manner. OsARF7 exhibits transcriptional activation activity, and its overexpression hyperactivates target genes such as the key fertility-related gene *ubiquitin-fusion ribosomal protein L40 4* and *heat shock protein 70-4*. Identification of the osphasiR1_1–OsAGO2–*OsARF7* regulatory module fills a critical gap in the phasiRNA pathway and expands our understanding of phasiRNA-mediated gene regulation, providing new insight into the molecular mechanisms that control male fertility in monocots.

Key words: phasiRNA, AGO, rice, male fertility, ARF, transcriptional activation

Shao L., Qiu K., Xu R., Zhou J., Yang J., Liu J., Yang Y., Li L., Xie B., Wang Y., Li J., Liu X., Yuan M., Yang Y., Li Y., Wei P., Jiang H., Qi Y., and Jiang P. (2026). A phased small interfering RNA acts in an OsAGO2-dependent manner to mediate *OsARF7* cleavage and promote male fertility in rice. Plant Comm. **7**, 101788.

INTRODUCTION

Plant small RNAs (sRNAs) are a class of short non-coding RNA molecules, typically 21–24 nucleotides (nt) in length (Borges and Martienssen, 2015; Chen and Kim, 2024). On the basis of differences in their precursor structures and processing components, they are classified into microRNAs (miRNAs) and small interfering RNAs (siRNAs), with the latter including heterochromatic siRNAs, *trans*-acting siRNAs (tasiRNAs), and phased siRNAs (phasiRNAs) (Borges and Martienssen, 2015; Fang and Qi, 2016). sRNAs are selectively loaded into distinct ARGONAUTE (AGO) proteins on the basis of their length and 5′ terminal nucleotide in order to fulfill their functions (Mi et al., 2008; Borges and Martienssen, 2015). For instance, 21-nt sRNAs with a 5′ U are preferentially loaded into AGO1, those with a 5′ C into AGO5, and those with a 5′ A into AGO2 (Mi et al., 2008; Borges and Martienssen, 2015). These 21-nt sRNAs mainly mediate the AGO-dependent cleavage of target RNAs, a process that depends on their high base complementarity. This cleavage is specifically catalyzed at the phosphodiester bond of the target RNA that lies between the two nucleotides complementary to the 10th and 11th nucleotides of the sRNA (Borges and Martienssen, 2015; Yu et al., 2025). By contrast, 24-nt sRNAs are predominantly associated with AGO4 and help to mediate DNA

This is an open access article under the CC BY license (http://creativecommons.org/licenses/by/4.0/).

methylation via the plant-specific RNA-directed DNA methylation pathway (Borges and Martienssen, 2015).

phasiRNAs and tasiRNAs are siRNAs whose precursors are cleaved in a phased manner (Toriba et al., 2010; Zhan and Meyers, 2023). In monocots, phasiRNAs are specifically expressed in the anthers and are closely associated with male fertility, thereby attracting significant research attention (Yu et al., 2018; Liu et al., 2020; Zhan and Meyers, 2023). The pathway of phasiRNA biogenesis is well characterized. This process initiates with transcription of *PHAS* loci by RNA polymerase II to generate single-stranded phasiRNA precursors (Liu et al., 2020). These precursors are then cleaved by AGO1, which is guided by miR2118 or miR2275, converted into fully complementary double-stranded RNAs by RNA-dependent RNA polymerase 6 (RDR6), and ultimately processed in a phased manner by Dicer-like 4 (DCL4) or DCL3b/DCL5 to generate arrays of 21- or 24-nt phasiRNAs (Liu et al., 2020). Like other sRNA species, mature phasiRNAs must be loaded into specific AGO proteins to perform their functions. Studies have revealed that 21-nt phasiRNAs with a 5′ C are preferentially loaded into the germline-specific AGO5 to mediate cleavage of downstream target genes, whereas those with a 5′ U associate with AGO1d to facilitate their translocation from somatic cells to male germ cells (Komiya et al., 2014; Jiang et al., 2020; Zhang et al., 2020; Tamotsu et al., 2023). Knockout of key components in the phasiRNA pathway, such as miR2118, *AGO1*, *RDR6*, *DCL4*, *DCL5,* and *AGO5,* results in male sterility (Nonomura et al., 2007; Song et al., 2012; Araki et al., 2020; Teng et al., 2020; Zhan, 2020; Zhang et al., 2021; Shi et al., 2022; Tamotsu et al., 2023; Bélanger et al., 2025). However, although thousands of *PHAS* loci and many more distinct phasiRNAs have been identified, the specific phasiRNAs that regulate male fertility remain to be determined. In addition, *PHAS* loci and phasiRNAs are not conserved between rice and maize (Lian et al., 2025), which has fueled concerns about whether individual phasiRNAs possess specific biological functions.

In *Arabidopsis thaliana*, AtAGO2 binds to the 5′-A miR393b* to cleave *Membrin 12* transcripts, thereby regulating disease resistance responses (Zhang et al., 2011b). Loss of *OsAGO2* function in rice reduces DNA methylation levels in the promoter region of *hexokinase 1* (*OsHXK1*), a gene involved in the reactive oxygen species (ROS) pathway, ultimately increasing ROS levels in anthers and causing severe defects in male fertility (Zheng et al., 2019). However, whether rice OsAGO2 ensures male fertility through 21-nt phasiRNA–mediated cleavage of downstream targets has not been investigated.

Auxin response factors (ARFs) bind to auxin response elements in the promoters of auxin-responsive genes, thereby activating or repressing their expression (Guilfoyle and Hagen, 2007). Genes from the *ARF* family regulate root development in both *Arabidopsis* and rice (Inukai et al., 2005; Wilmoth et al., 2005). In addition, some specific *ARF*s modulate plant height, leaf angle, and tiller angle in rice (Attia et al., 2009; Sakamoto et al., 2013). As well as regulating vegetative growth, *ARF*s also control reproductive development. For instance, knockout of *OsARF2* or overexpression (OE) of *OsARF18* in rice results in closed spikelets and failure of anther dehiscence (Zhao et al., 2022). miRNAs have also been reported to ensure fertility by targeting *ARF*s. In *Arabidopsis*, miR160 mediates cleavage of *ARF17* transcripts to ensure anther and pollen development (Wang et al., 2017). Similarly, miR167 mediates cleavage of *ARF6* and *ARF8* transcripts in *Arabidopsis* and tomato, and mutations in the *ARF6* or *ARF8* target sequences that are complementary to miR167 lead to increased *ARF* expression, which impairs both female and male fertility (Wu et al., 2006). In *Arabidopsis*, the abaxial determinants *ETTIN* and *ARF4* are silenced by *TAS3*-derived tasiRNAs (Allen et al., 2005). Notably, defects of *SHOOTLESS2*/*OsRDR6* in rice lead to reduced levels of tasiRNA-directed cleavage of *ARF* and *ETT* and produce aberrant anther patterning, implying that a tasiRNA–*ARF* module operates in monocot male development (Toriba et al., 2010). Whether phasiRNAs can also regulate male fertility in monocots by targeting *ARF* genes warrants investigation.

In this study, we identified a functional 21-nt phasiRNA (designated osphasiR1_1) that positively regulates male fertility in rice by directing the cleavage of *OsARF7* transcripts in an OsAGO2-dependent manner. These findings address the long-standing question of whether individual reproductive-specific phasiRNAs possess biological functions and further expand known phasiRNA functional pathways by revealing an OsAGO2-dependent module.

RESULTS

Loss of osphasiR1_1 function reduces male fertility

In our previous work, we identified 543 21-nt phasiRNAs that mediate the cleavage of target genes in male germ cells of rice (Jiang et al., 2020). To further identify functionally conserved 21-nt phasiRNAs across diverse rice varieties, we screened those with detectable target cleavage signals in Nipponbare (*japonica*), Zhongxian 3037 (3037, *indica*), and Huanghuazhan (*indica*) whose target degradome signals all ranked in the top 20. A total of 12 phasiRNAs met the criteria (Supplemental Table 1). Among these, 10 phasiRNAs possess a 5′ C, which is consistent with the established functional model in which 21-nt phasiRNAs exert their roles through AGO5. Unexpectedly, one phasiRNA with a 5′ A (21PHAS466_247-) was also identified in our screening results (Supplemental Table 1). Given that 5′-A-containing sRNAs specifically associate with AGO2, this result suggested that this phasiRNA may function via a novel pathway independent of AGO5 and thus drew our attention (the phasiRNA locus and the specific 5′-A phasiRNA are referred to as *OsPHAS1* and osphasiR1_1 hereafter).

To determine whether osphasiR1_1 has biological functions in the regulation of male fertility, we performed large-fragment knockout of the *PHAS* locus using CRISPR–Cas9, obtaining one line (*osphas1-1*) with a 69-bp insertion and a 731-bp deletion (Figure 1A and 1B). Because large-fragment knockout also leads to the loss of other phasiRNAs derived from *OsPHAS1*, we also knocked down osphasiR1_1 specifically using the short tandem target mimic (STTM) technique (Figure 1C) (Yan et al., 2012). We collected anthers at the meiosis stage from T_2 seedlings of STTM transgenic-positive lines and detected the expression level of osphasiR1_1 by stem-loop quantitative reverse-transcription PCR (RT–qPCR) (Panwalkar et al., 2022). Two STTM knockdown lines with significantly reduced osphasiR1_1

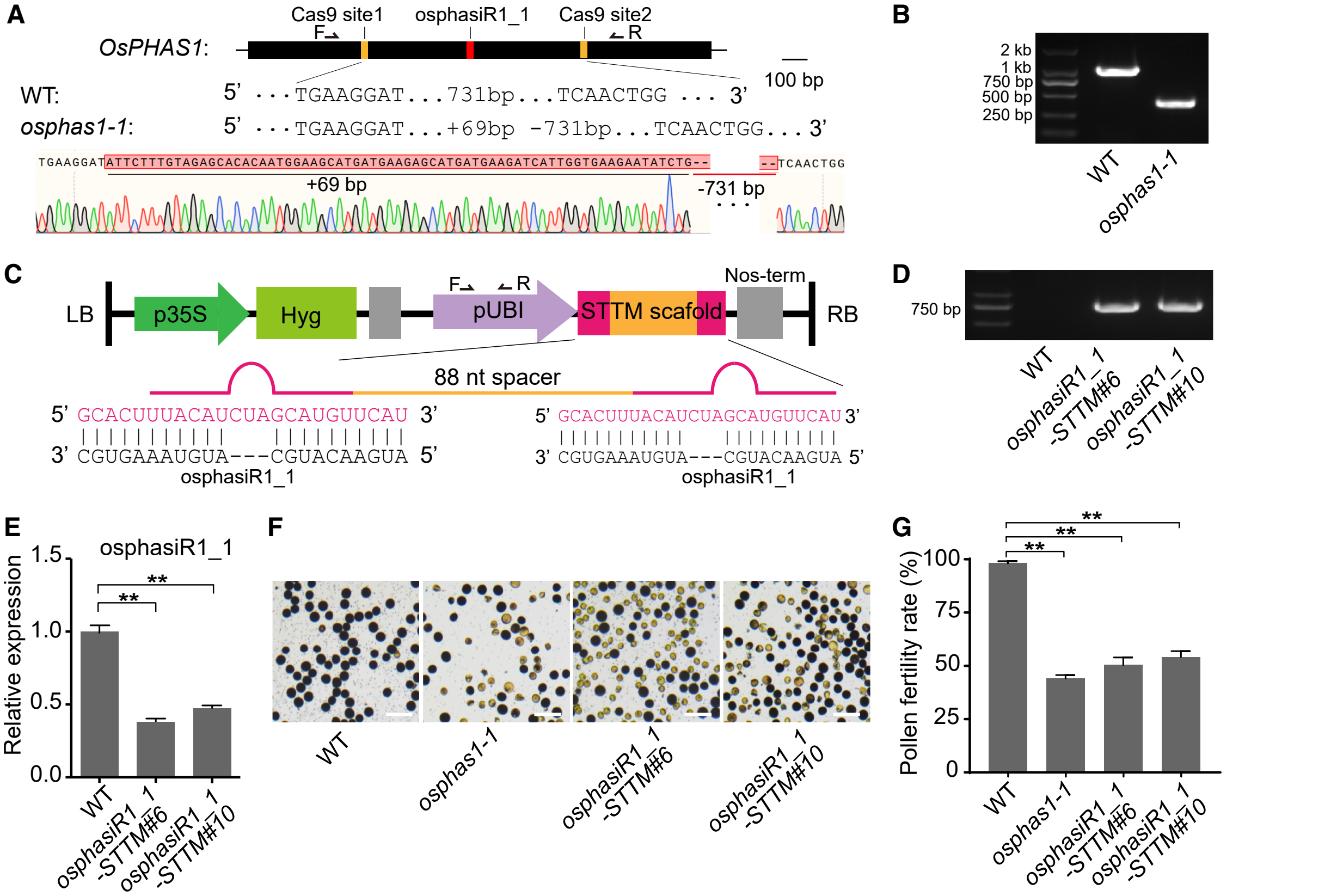

Figure 1. Loss of osphasiR1_1 function reduces male fertility.

(A) Schematic of the *OsPHAS1* gene structure and sequence of the *osphas1-1* mutant. F and R primers were used for genotyping.

(B) Genotyping of the *osphas1-1* mutant.

(C) Schematic of the STTM structure. F and R primers were used for genotyping of transgenic-positive lines.

(D) Genotyping of *osphasiR1_1-STTM#6* and *osphasiR1_1-STTM#10* transgenic-positive lines.

(E) Expression levels of osphasiR1_1 in STTM lines identified by stem-loop RT–qPCR. Three biological replicates (n = 3) of each line were used, and significant differences were identified using the t-test, $^{**}P < 0.01$.

(F and G) Pollen from mutants stained with iodine–potassium iodide (I_2–KI) and statistics for pollen fertility. Ten plants per line were used; t-test, $^{**}P < 0.01$. Bar: 200 μm.

expression were identified: *osphasiR1_1-STTM#6* and *osphasiR1_1-STTM#10* (Figure 1D and 1E). To determine whether osphasiR1_1 positively regulates male fertility, we performed iodine–potassium iodide staining of mature pollen from the mutant materials. As expected, both knockout and knockdown lines exhibited significantly reduced pollen fertility (Figure 1F and 1G), and the seed-setting rate was also reduced in the mutants (Supplemental Figure 1A and 1B). There were no significant differences in plant vegetative growth between the mutants and the wild type (WT) (Supplemental Figure 1C). These results reveal that osphasiR1_1 specifically ensures male fertility in rice.

OsphasiR1_1 promotes male fertility through direct cleavage of *OsARF7* transcripts

Degradome sequencing data from our previous work revealed that osphasiR1_1 mediates the cleavage of *OsARF7* transcripts. The rice ARF family comprises 25 members, among which *OsARF7* shares a close phylogenetic relationship with *OsARF9* and *OsARF1* (Supplemental Figure S2A). However, sequence analysis demonstrated that osphasiR1_1 exhibits robust complementarity only to the mRNA sequence of *OsARF7* (Figure 2A and 2B; Supplemental Figure 2B and 2C). To confirm that osphasiR1_1 specifically mediates *OsARF7* cleavage, we collected male germ cells at the meiosis stage from the WT and the *osphas1-1* mutant. Consistent with our previous degradome data, a 5′ rapid amplification of cDNA ends (RACE) assay showed that the 5′ base of the inserted fragments from most positive clones mapped to the predicted cleavage site mediated by osphasiR1_1 in the WT but not in the *osphas1-1* mutant line (Figure 2B). The expression level of *OsARF7* was also significantly higher in the *osphas1-1* and *osphasiR1_1 STTM* lines (Figure 2C). These results indicate that osphasiR1_1 represses the expression of *OsARF7* by directing the cleavage of its transcripts.

We next asked whether osphasiR1_1 ensures male fertility by cleaving *OsARF7*. Because plant sRNA-mediated target recognition relies on sequence complementarity, we mutated the sequences in the 3′ UTR of *OsARF7* that are complementary to osphasiR1_1, then overexpressed mutated *OsARF7* ($OsARF7^{mu}$) or WT *OsARF7* ($OsARF7^{WT}$) under the control of its native promoter (Figure 2D). We obtained 20 $OsARF7^{mu}$*-OE* T_0 lines

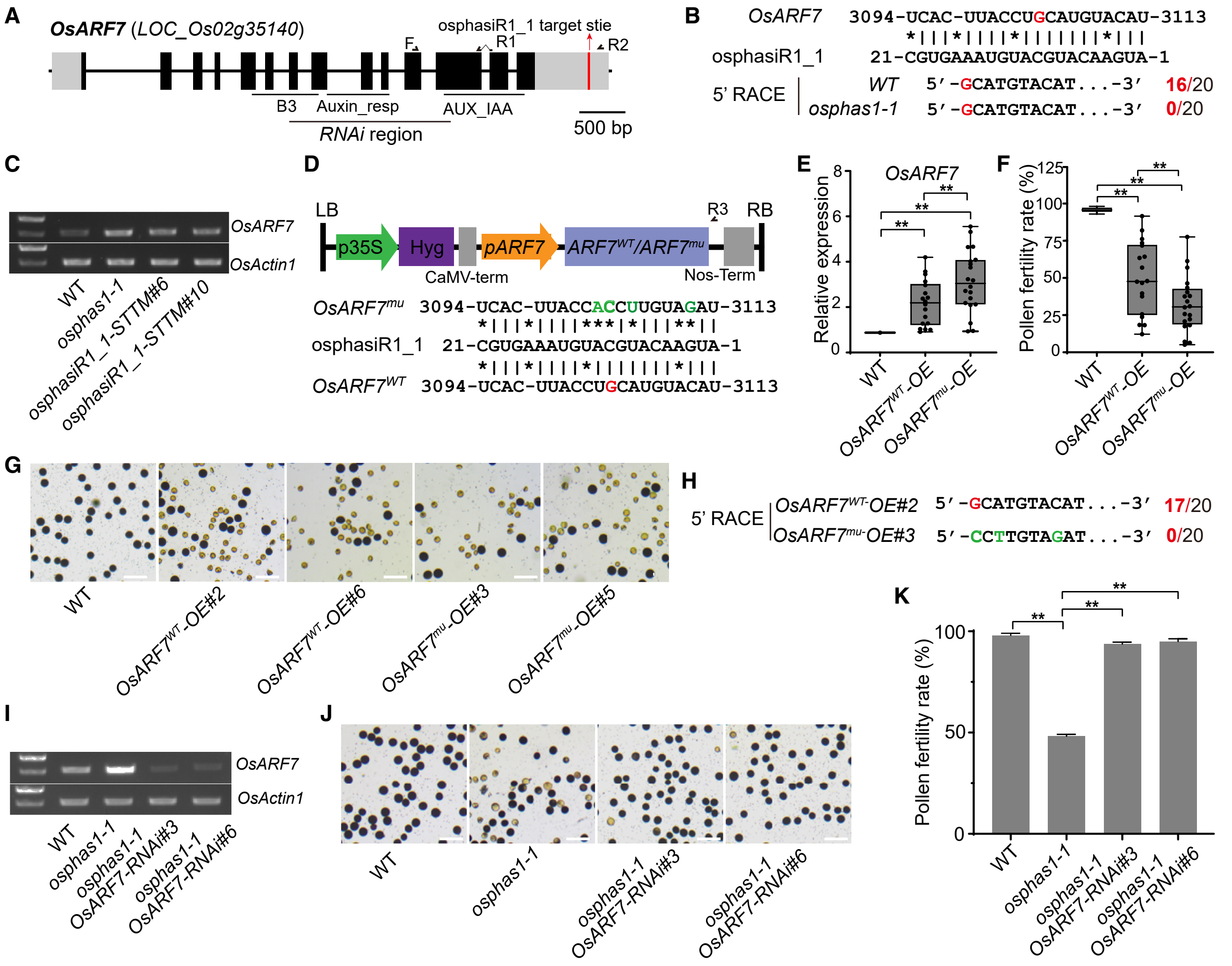

Figure 2. OsphasiR1_1 promotes male fertility through direct cleavage of *OsARF7* transcripts.
(A) Gene structure of *OsARF7*. F and R1 primers were used for semi-quantitative PCR, and an R2 primer was used for 5′ RACE in mutants.
(B) Top: pairing of osphasiR1_1 with its target sequence. Bases in red represent the 5′ base of the cleaved products of *OsARF7*. Bottom: 5′ RACE analysis of the *osphas1-1* mutant and WT. Twenty clones from each material were sequenced; numbers in bold red represent positive clones that matched the predicted cleavage site.
(C) Semi-quantitative PCR analysis of *OsARF7* expression. *OsActin1* was used as the internal control.
(D) Schematic of vectors and sequences used for *OsARF7* overexpression (OE). Mutated bases are shown in green. The R3 primer was used for 5′ RACE analysis in *OsARF7^{WT}-OE* and *OsARF7^{mu}-OE* lines.
(E and F) *OsARF7* expression levels and pollen fertility in OE rice at the T_2 generation. At least three homozygous plants of each T_2 line were used, and each black solid dot indicates the average value of one line. *t*-test, $^{**}P < 0.01$ and $^{*}P < 0.05$.
(G) I_2–KI staining of pollen from each of two lines of *OsARF7^{WT}-OE* and *OsARF7^{mu}-OE*. Bar: 200 μm.
(H) 5′ RACE analysis of *OsARF7^{WT}-OE#3* and *OsARF7^{mu}-OE#2*.
(I) Semi-quantitative PCR analysis of *OsARF7* expression. *OsActin1* was used as the internal control.
(J and K) I_2–KI staining and statistics for pollen fertility of *osphas1-1* and *osphas1-1 OsARF7-RNAi* double mutants. Bar: 200 μm.

and 18 *OsARF7^{WT}-OE* T_0 lines and collected anthers from T_2 homozygous plants at the meiosis stage for RT–qPCR analysis. As expected, *OsARF7* expression was significantly higher in *OsARF7^{mu}-OE* lines, and their pollen fertility and seed-setting rate were significantly reduced (Figure 2E–2G; Supplemental Figure 3). Consistent with these results, 5′ RACE assays using PCR primers specific to vector sequences and 5′ adapter sequences showed that the 5′ ends of most positive clones in *OsARF7^{WT}-OE* lines mapped to the target cleavage site, whereas the 5′ ends in *OsARF7^{mu}-OE* lines did not, indicating that osphasiR1_1 can direct the cleavage of *OsARF7^{WT}* but not *OsARF7^{mu}* (Figure 2H). These results revealed that osphasiR1_1 regulates male fertility by mediating the cleavage of *OsARF7*.

To confirm that osphasiR1_1 and *OsARF7* function in the same pathway to regulate male fertility, we performed RNAi-mediated knockdown of *OsARF7* in the *osphas1-1* background. Semi-quantitative RT–PCR (semi-RT–PCR) of anthers at the meiosis stage showed that *OsARF7* expression was significantly lower and that pollen fertility and seed-setting rate were significantly

restored in the *osphas1-1 OsARF7-RNAi* double mutants compared with the *osphas1-1* single mutant (Figure 2I–2K). We noticed that *OsARF7* expression in the double mutants was also significantly lower than that in the WT control (Figure 2I). This result suggested that reduced *OsARF7* expression does not impair fertility. To confirm this, we ordered seeds of the *osarf7-1* knockout mutant from BIOGLE GeneTech (http://biogle.cn/) and identified homozygous seedlings with a shifted open reading frame and a premature stop codon (Supplemental Figure 4A). As expected, there was no significant difference in pollen fertility between *osarf7-1* and the WT control (Supplemental Figure 4B and 4C).

OsphasiR1_1-directed cleavage of *OsARF7* is dependent on OsAGO2

It is well established that 21-nt phasiRNAs function by associating with OsAGO5c. However, osphasiR1_1 harbors a 5′ A, suggesting that it should be loaded into AGO2 rather than AGO5. AGO regulates sRNA abundance in two main ways. One is biogenesis dependent and typically involves the triggering of siRNA biogenesis; examples include the biogenesis of phasiRNAs triggered by AGO1 and the biogenesis of tasiRNAs triggered by AGO7. The other is independent of biogenesis: AGO binding protects the sRNA from exonucleolytic degradation after 5′-nt- and length-based recognition, and loss of the cognate AGO therefore accelerates sRNA degradation. Our published sRNA sequencing data indicated that osphasiR1_1 abundance was not reduced in the *osago5c-4* mutant (Jiang et al., 2020), suggesting that osphasiR1_1 may function through an AGO5-independent pathway. To confirm this possibility, we generated *OsAGO2* and *OsAGO5c* knockout mutants in the Zhonghua 11 (ZH11) background using CRISPR–Cas9 (Figure 3A; Supplemental Figure 5). Consistent with previous studies, *osago2-3* and *osago2-4* showed severe defects in male fertility, and *osago5c-5* was male sterile (Supplemental Figure 6A and 6B). We then performed stem-loop RT–qPCR to assess the abundance of osphasiR1_1 in anthers of *osago2 and osago5c mutants* at the meiosis stage. The expression level of osphasiR1_1 was significantly reduced in the *osago2-3* and *osago2-4* mutants, but no obvious change was observed in the *osago5c-5* mutant (Figure 3B). Subsequent northern blot analysis yielded consistent results (Figure 3C). These findings indicate that osphasiR1_1 associates with OsAGO2 but not with OsAGO5c. In addition, 5′ RACE assays showed that in *osago2* mutants, the 5′ ends of inserted fragments from positive clones did not map to the target cleavage site, whereas those from *osago5c-5* did (Figure 3D). Concomitantly, *OsARF7* expression was significantly upregulated in *osago2* mutants but unchanged in the *osago5c* mutant (Figure 3E; Supplemental Figure 6C). All of these data show that osphasiR1_1 directs the cleavage of *OsARF7* through association with OsAGO2.

To genetically verify that OsAGO2 regulates male fertility through *OsARF7*, we knocked down *OsARF7* in the *osago2-3* background (Figure 3F). The pollen fertility and seed-setting rate of the *osago2-3 OsARF7-RNAi* double mutant were significantly restored compared with those of *osago2-3* (Figure 3G and 3H; Supplemental Figure 7). Together, these results indicate that osphasiR1_1 directs OsAGO2 to cleave *OsARF7* transcripts, thus ensuring male fertility in rice.

Knockout of osphasiR1_1 or overexpression of *OsARF7* results in abnormal development of male germ cells

To further dissect the cytological basis for osphasiR1_1-mediated regulation of male fertility, we collected rice spikelets 2–8 mm in length from ZH11, *osphas1-1*, and *OsARF7^{mu}-OE#3,* in which the anther length was 0.3–2.2 mm. The male germ cells inside the anther were at various developmental stages, from early meiosis to mature pollen, as described previously (Zhang et al., 2011a). These spikelets were fixed and paraffin-sectioned; anther developmental stages were first inferred from spikelet length and anther length and then verified by microscopic examination according to previously reported descriptions (Zhang et al., 2011a). At the S7 stage, germ cells of *osphas1-1* and *OsARF7^{mu}-OE#3* exhibited obvious morphological abnormalities, such as extensive vacuolation. At the S8 and S9 stages, shriveled and collapsed germ cells were observed in *osphas1-1* and *OsARF7^{mu}-OE#3*, and at the S12 stage, anthers from *osphas1-1* and *OsARF7^{mu}-OE#3* exhibited shriveling and abortion of mature pollen (Figure 4A). Because 21-nt phasiRNAs were abundant in germ cells at the meiosis stage, we used 4′,6-diamidino-2-phenylindole staining to examine chromosome behavior in pollen mother cells of *osphas1-1* and *OsARF7^{mu}-OE*#3 anthers. At the diakinesis stage, WT chromosomes were highly condensed to form 12 bivalents, whereas the *osphas1-1* pollen mother cells exhibited chromosome adhesion and the formation of incomplete bivalents (Figure 4B). During anaphase I, homologous chromosomes segregated normally in the WT, but *osphas1-1* exhibited chromosome bridges. These defects generated aberrant tetrads with multiple micronuclei and chromosomal fragments after cytokinesis (Figure 4B). Comparable meiotic abnormalities were observed in *OsARF7^{mu}-OE*#3 (Figure 4B), indicating that elevated *OsARF7* expression phenocopies the *osphas1-1* meiotic phenotype.

OsARF7 is precociously derepressed in *osphas1-1* anthers

Premature or delayed expression of key genes can cause developmental abnormalities and reduced fertility during anther development. To determine whether the osphasiR1_1–*OsARF7* circuit is temporally regulated, we monitored the expression dynamics of osphasiR1_1 and *OsARF7* throughout anther development. Anthers were harvested from spikelets of 2, 4, and 6 mm in length and from 8-mm spikelets before and after panicle shooting (referred to as M2, M4, M6, M8E, and M8L anthers hereafter) (Figure 5A). Stem-loop RT–qPCR showed that osphasiR1_1 expression peaked in M2 anthers of ZH11 and declined thereafter, becoming undetectable at M8E (Figure 5B). By contrast, *OsARF7* transcript levels rose steadily from M2 to M8E and subsequently declined at M8L (Figure 5C). In *osphas1-1* anthers, osphasiR1_1 expression was undetectable at all stages, whereas *OsARF7* expression was significantly higher in early-stage anthers compared with that in ZH11 (Figure 5B and 5C). These findings indicate that the loss of osphasiR1_1 function leads to premature derepression of *OsARF7* in early-stage anthers, which may impair male fertility. To verify the tissue expression patterns of osphasiR1_1 and *OsARF7*, we performed RNA *in situ* hybridization with ZH11 anthers from 2-mm spikelets. The results showed that osphasiR1_1 expression was relatively enriched in the tapetum and germ cells, whereas *OsARF7* was constitutively expressed in the anthers (Figure 5D).

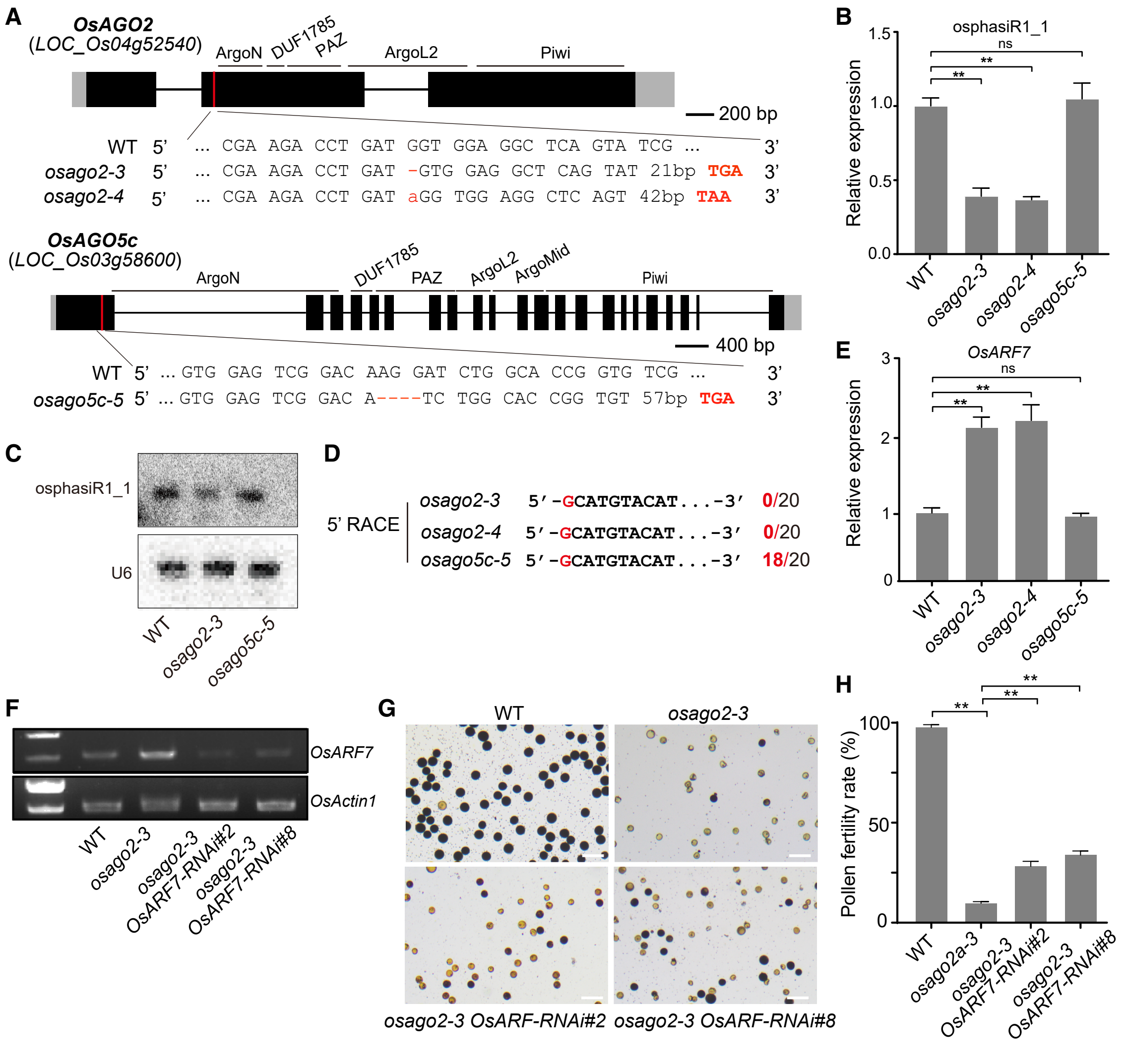

Figure 3. OsphasiR1_1-directed cleavage of *OsARF7* transcripts is dependent on OsAGO2.
(A) Gene structures of *OsAGO2* and *OsAGO5c* and characterization of their knockout mutants. The red vertical lines indicate the Cas9 target site.
(B and C) Analysis of osphasiR1_1 levels in *osago2* and *osago5c* mutants via stem-loop RT–qPCR and RNA northern blot. $N = 3$ biological replicates; *t*-test, $^{**}P < 0.01$.
(D) 5′ RACE analysis of cleaved *OsARF7* transcripts in *osago2* and *osago5c* mutants.
(E) RT–qPCR analysis of *OsARF7* expression in *osago2* and *osago5c* mutants. $N = 3$ biological replicates; *t*-test, $^{**}P < 0.01$.
(F–H) Semi-quantitative PCR analysis of *OsARF7* expression, I_2–KI staining of pollen, and statistics for pollen fertility in *osago2-3* and *osago2-3 OsARF7-RNAi* double mutants. Two anthers from 10 seedlings of each mutant were used. *t*-test, $^{**}P < 0.01$. Bar: 200 μm.

OsARF7 has transcriptional activation activity and promotes the expression of $Ub_{L40}4$ and *cHSP70-4* in anthers

As a canonical ARF, OsARF7 contains a B3-type DNA-binding domain at the N terminus and Auxin_resp and AUX_IAA domains at the C terminus. To dissect its transcriptional properties, we cloned the full-length coding sequence and consecutive truncations of *OsARF7* into pGBKT7 and transformed the resulting constructs into yeast strain AH109 (Figure 6A and 6B). Yeast cells carrying pGBKT7-OsARF7 (full-length) or the OsARF7 C-terminal fragment (OsARF7C) proliferated on SD/–His/–Trp/–Ade medium, whereas transformants harboring pGBKT7-OsARF7N did not, indicating that OsARF7 likely possesses transcriptional activation activity through the domain that resides in the C-terminal region (Figure 6B). To confirm the transactivation activity of OsARF7, we performed a transient dual-luciferase assay in tobacco leaves. We fused OsARF7 to the GAL4 DNA-binding domain under the CaMV 35S promoter in pCAMBIA1300 and co-expressed it with a *GAL4*-responsive firefly luciferase (LUC) reporter (Figure 6C). Tobacco leaves co-transformed with OsARF7 and LUC reporter vectors showed a clear LUC signal, whereas the control did not, and the presence of OsARF7 increased relative LUC activity approximately 10-fold relative to that of the vector control ($p < 0.01$), confirming that OsARF7 functions as a transcriptional activator *in vivo* (Figure 6D and 6E).

To identify genes transcriptionally activated by OsARF7, we profiled M2 anthers of ZH11 and *OsARF7*^{mu}*-OE*#3 by RNA sequencing (RNA-seq). Differentially expressed genes were identified using thresholds of $|\log_2 FC| \geq 1$ (2-fold change) and a false

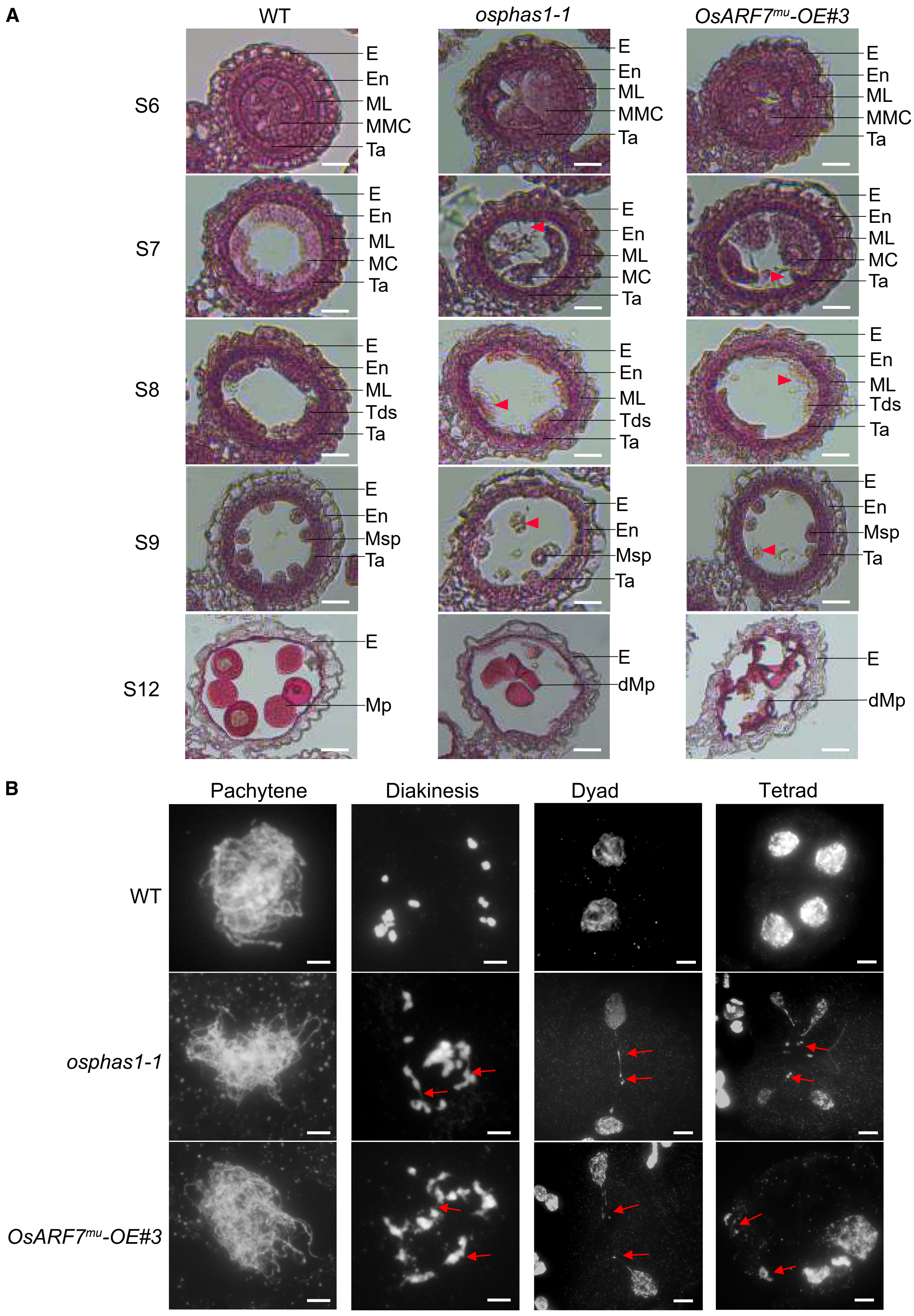

(legend on next page)

discovery rate < 0.05. Volcano plots revealed a strong bias toward upregulated genes, and 65 of the top 100 differentially expressed genes were upregulated (Figure 6F; Supplemental Figure 8). These results are consistent with the activator function of OsARF7. Notably, many of these upregulated genes have been reported to participate in the regulation of male fertility in rice. For example, *ubiquitin-fusion ribosomal protein L40 4* (*$Ub_{L40}4$*), a direct target of *thermosensitive genic male sterile 5*, causes male sterility when overexpressed in rice (Zhou et al., 2014). Likewise, *heat shock protein 70-4* (*cHSP70-4*), a downstream target of the anther-enriched *reduced heat stress tolerance* (*OsRHS*) gene, severely reduces rice seed set upon OE (Mao et al., 2025). In addition, knockouts of *ATP-binding cassette G26* (*OsABCG26*), *glycerol-3-phosphate acyltransferase 3* (*OsGPAT3*), *undeveloped tapetum 1* (*Udt1*), and *pectin methylesterase inhibitor 12* (*OsPMEI12*) all result in male sterility (Jung et al., 2005; Chang et al., 2016; Sun et al., 2018; Li et al., 2022).

We verified the expression of these genes by RT–qPCR, and the results were consistent with the RNA-seq data, with all six genes significantly upregulated in anthers of *$OsARF7^{mu}$-OE#3* and *$OsARF7^{mu}$-OE#5* relative to ZH11 (Figure 6G and 6H; Supplemental Figure 9). To further confirm that OsARF7 directly regulates the expression of these genes, we selected the 1.5-kb sequences upstream of the start codon of *$Ub_{L40}4$* and *cHSP70-4* (whose OE reduces fertility) as their promoters. Bioinformatics analysis showed that both promoters contained multiple auxin response DNA elements (Boer et al., 2014) (Figure 6I and 6J). We introduced their full-length promoters and different promoter fragments into the LUC reporter system and co-transformed these constructs into rice protoplasts with 1300-35S-OsARF7 (Figure 6I and 6J). OsARF7 significantly activated LUC expression driven by the full-length promoters compared with that observed in the control group co-transformed with the *1300-35S empty vector* (Figure 6K and 6L). Among the truncated promoters, only those containing ARF-binding elements mediated OsARF7-induced LUC activation (Figure 6K and 6L). Collectively, these results indicate that OsARF7 can promote the expression of the target genes *$Ub_{L40}4$* and *cHSP70-4*. The loss of osphasiR1_1 may lead to OE of these genes, thereby reducing male fertility.

DISCUSSION

It has been reported that 21-nt phasiRNAs are loaded into AGO5 to mediate the cleavage of target genes. However, whether specific 21-nt phasiRNAs possess biological functions remains unknown. OsphasiR1_1 is 21 nt in length and carries a 5′ A, a signature preferentially bound by AGO2, whereas AGO5c primarily loads 5′-C-containing sRNAs. Consistent with this specificity, osphasiR1_1 levels and *OsARF7* expression remained unchanged in *osago5c* mutants, whereas osphasiR1_1 abundance dropped and *OsARF7* transcripts accumulated in *osago2 mutants*, and the fertility defects of *osago2* and *osphas1-1* were partially rescued by knockdown of *OsARF7*. Collectively, these data indicate that osphasiR1_1 is loaded into OsAGO2 rather than OsAGO5c; this loading protects the phasiRNA from degradation and enables *OsARF7* cleavage. In this study, we not only identified a functional 21-nt phasiRNA (osphasiR1_1) but also demonstrated how it regulates male fertility by mediating OsAGO2-dependent cleavage of the target gene *OsARF7* (Figure 7). This work fills a gap in the phasiRNA pathway, linking the biogenesis of 21-nt phasiRNAs to their functional output and demonstrating that 21-nt phasiRNAs can function through AGO proteins other than AGO5. The Komiya group reported that 5′-U 21-nt phasiRNAs can associate with AGO1 and that such an interaction contributes to the translocation of phasiRNAs from somatic cells to germ cells. In addition to transporting 5′-U 21-nt phasiRNAs, AGO1 may also mediate the cleavage of target genes via these phasiRNAs to regulate fertility, a possibility that remains to be explored. phasiRNAs with 5′ U and 5′ G were also shown to direct the cleavage of target transcripts in male germ cells in our previous study (Jiang et al., 2020). Whether anther-expressed AGO proteins other than AGO5 and AGO2 can also regulate male fertility via phasiRNAs warrants further investigation.

Notably, *osago2* mutants exhibit a severe male-sterile phenotype, whereas the sterile phenotype caused by osphasiR1_1 deficiency is relatively mild. First, OsAGO2 is likely to regulate male fertility through both the 21-nt phasiRNA pathway described here and the previously reported *OsHXK1*-ROS pathway. Thus, loss of function of osphasiR1_1 only partially impairs the function of OsAGO2. Second, previous degradome analysis identified additional 5′-A 21-nt phasiRNAs that mediate the cleavage of target genes in anthers and male germ cells (Jiang et al., 2020; Zhang et al., 2020). These 5′-A 21-nt phasiRNAs may also regulate male fertility through OsAGO2, such that the phenotype caused by loss of function of a specific 5′-A phasiRNA is milder than that of *osago2*. In addition, the sterile phenotype of *$OsARF7^{mu}$-OE#3* is more severe than that of *osphas1-1*. As described previously, when *OsARF7*-overexpressing lines were identified, the OE level of *OsARF7* was negatively correlated with male fertility. We performed RT–qPCR analysis of *OsARF7* expression in M2–M8L anthers from *$OsARF7^{mu}$-OE#3* and *osphas1-1* and found that relative *OsARF7* expression in anthers was higher in *$OsARF7^{mu}$-OE#3* than in *osphas1-1,* even at the M8L stage, at which osphasiR1_1 is not expressed (Supplemental Figure 10). This is likely the reason why the fertility phenotype of *osphas1-1* is less severe than that of *$OsARF7^{mu}$-OE#3*.

Interestingly, OE of *OsARF7* resulted in reduced male fertility, but knockout of *OsARF7* did not. Phylogenetic analysis revealed that *OsARF7* shares a close phylogenetic relationship with *OsARF1* and *OsARF9*, and these three genes likely show functional redundancy in the regulation of male fertility. The mechanisms by which auxin regulates rice male fertility remain largely unknown;

Figure 4. Cytological defects of germ cells in *osphas1-1* and *$OsARF7^{mu}$-OE#3* anthers.

(A) Paraffin sections of anthers from the S6 stage to the mature pollen stage. Extensive vacuolation at the S7 stage and shriveled or collapsed germ cells at the S8/S9 stage are indicated with red triangles. E, epidermis; En, endothecium; ML, middle layer; MMC, microspore mother cell; MC, meiocyte; Msp, microspore parietal cell; Ta, tapetum; Tds, tetrads; Mp, mature pollen; dMp, defective mature pollen. Bar: 20 μm.

(B) 4′,6-diamidino-2-phenylindole (DAPI) staining of male meiocytes. Chromosome adhesion and the formation of incomplete bivalents at the diakinesis stage, chromosome bridges in dyads, and multiple micronuclei and chromosomal fragments in tetrads are indicated with red arrows. Bar: 10 μm.

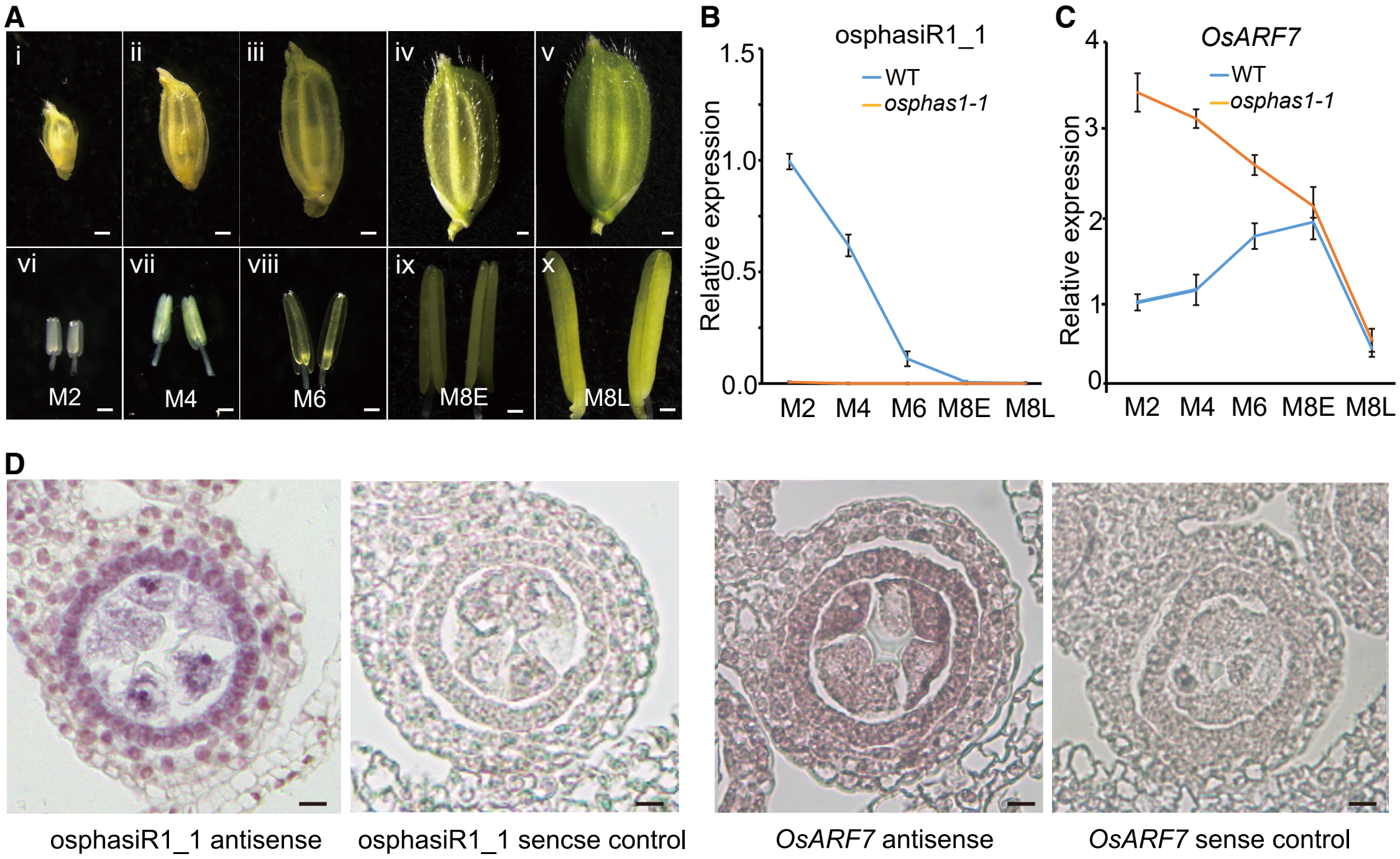

Figure 5. Expression patterns of osphasiR1_1 and *OsARF7* during anther development.

(A) Sampling scheme of anthers from spikelets of different lengths. M2/4/6/8 represent male organ from 2/4/6/8 mm spikelet, 8E and 8L represent 8 mm spiklet from panicles before- and after-shooting **(i–v)** Bar: 0.5 mm. **(vi–x)** Bar: 0.2 mm.

(B) Stem-loop RT–qPCR of osphasiR1_1 (n = 3 biological replicates).

(C) RT–qPCR of *OsARF7* (n = 3 biological replicates).

(D) RNA *in situ* hybridization of osphasiR1_1 and *OsARF7* in M2 anthers. Bar: 10 μm.

simultaneous knockout of these three genes and further investigation of these *ARF*s will help to address these questions.

METHODS

Plant materials and gene accession numbers

The rice cultivar ZH11 (*Oryza sativa* ssp. *japonica* var. ZH11) and the transgenic plants were grown in a field in Hefei, China (31°49′ N, 117°13′ E), from May to October. The daylength ranged from 12 to 13.5 h. The *osphas1-1*, *osago2-3*, *osago2-4*, and *osago5c-5* knockout mutants were generated using CRISPR–Cas9 and callus transformation in the lab. The *osarf7-1* mutant was ordered from BIOGLE GeneTech (http://biogle.cn/). Mutations in the transgenic plants were characterized by Sanger sequencing. Tillers at the proper stage were harvested to collect spikelets, anthers, and male germ cells (Zhang et al., 2011a; Jiang et al., 2020). The collected spikelets were used for paraffin sections; anthers for RT–PCR analysis, stem-loop qPCR, and northern blotting; and male germ cells for 5′-RACE.

The genomic position of *OsPHAS1* was described in our previous study (Jiang et al., 2020). The Rice Genome Annotation Project LOC IDs of key genes were *OsAGO2/LOC_Os04g52540*, *OsAGO5c/LOC_Os03g58600*, *OsARF7/LOC_Os02g35140*, *OsARF1/LOC_Os01g13520*, *OsARF9/LOC_Os04g36054*, *OsActin1/LOC_Os03g50885*, *Ub*$_{L40}$*4/LOC_Os09g31031*, *cHSP70-4/LOC_Os03g16920*, *OsABCG26/LOC_Os10g35180*, *OsPMEI12/LOC_Os03g01020*, *OsGPAT3/LOC_Os11g45400*, and *Udt1/LOC_Os07g36460*.

Plasmid construction and transformation

OE cassettes for *OsARF7*^{mu} and *OsARF7*^{WT} driven by the native promoter (2776 bp) were inserted into the *pCAMBIA1300* vector. The STTM vectors were constructed as described previously, with modifications (Yan et al., 2012). The STTM cassette was cloned into the *pCAMBIA1300* vector driven by the maize ubiquitin promoter. To construct the RNAi vector for *OsARF7*, a ~300-bp fragment of the cDNA sequence and its reverse complementary sequence were cloned into the *pUCCRNAi* vector, and the RNAi cassette was transferred into the *pCAMBIA1300* vector and driven by the maize ubiquitin promoter. All constructs were transformed into *Agrobacterium tumefaciens* strain EHA105 and then introduced into ZH11. All primers used in this study are listed in Supplemental Table 2.

Collection of anthers and male germ cells

Meiotic spikelets with anthers and germ cells at the meiosis stage were collected as described previously, with minor modifications (Jiang et al., 2020). Tillers whose flag leaf collar was about 5 cm below the collar of the penultimate leaf were chosen. Spikelets collected from the panicles were placed on a slide, and anthers were swiftly dissected using a needle, then flash-frozen in liquid nitrogen. To collect germ cells, a drop of 1× phosphate-buffered saline (PBS) was added immediately after dissection of the anthers using a needle. Gentle pressure was applied to the anthers to release germ cells from the anther walls. After the addition of one drop of 1× PBS, these cells were collected with capillary glass pipettes without taking any somatic cell debris. The collected anthers were ground in liquid nitrogen,

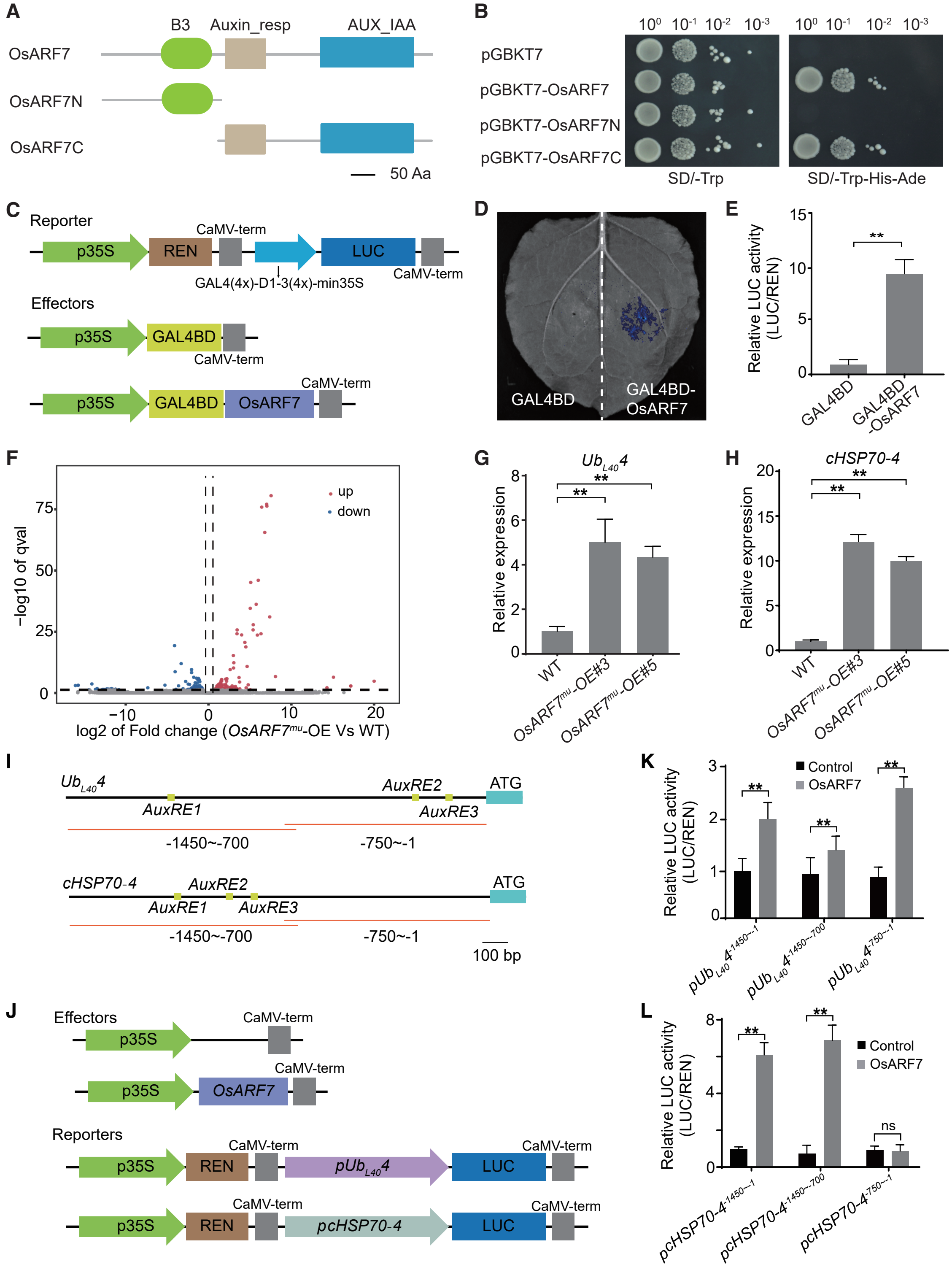

Figure 6. OsARF7 functions as a transcriptional activator and promotes the expression of $Ub_{L40}4$ and *cHSP70-4*.

(A) Schematic of full-length and truncated *OsARF7* fused to GAL4-BD in pGBKT7.

(B) Yeast growth on SD/−Trp (control) and SD/−Trp−His−Ade media.

(C) Schematic of effectors and reporters used in the dual-luciferase assay.

(D) LUC imaging of *Nicotiana benthamiana* leaves co-transformed with effector and reporter vectors at 48 h post infiltration.

(E) Relative LUC activity (n = 3 biological replicates; t-test, $^{**}P < 0.01$).

(legend continued on next page)

resuspended in 500 μl TRIzol reagent (Vazyme), and stored in 1.5-ml LoBind tubes at −80°C for future RNA extraction. The collected germ cells were washed twice with 1× PBS, resuspended in 100 μl TRIzol, and stored in 0.2-ml LoBind tubes at −80°C for future RNA extraction.

M2 and M4 anthers, including anthers from spikelets whose length was less than 4 mm, were collected from tillers whose flag leaf collar was about 5 cm below the collar of the penultimate leaf. M6 anthers, including anthers from spikelets whose length was 4–6 mm, were collected from tillers whose flag leaf collar was about 1 cm above the collar of the penultimate leaf. M8E and M8L anthers were collected from tillers whose panicles had not yet emerged or had fully emerged from the leaf sheath, respectively.

Semi-RT–PCR, RT–qPCR, and stem-loop RT–qPCR

Total RNA was extracted using TRIzol, and first-strand cDNA was reverse transcribed from 2 μg of total RNA. The HiScript III 1st Strand cDNA Synthesis Kit (Vazyme) with oligo-dT reverse-transcription primer was used for semi-RT–PCR and RT–qPCR. The miRNA 1st Strand cDNA Synthesis Kit (Vazyme) was used for stem-loop RT–qPCR, and reverse-transcription primers were designed as described previously (Panwalkar et al., 2022). Semi-quantitative RT–PCR (semi-RT–PCR) was performed using Hieff PCR Master Mix (Yeasen). RT–qPCR analyses were performed using AceQ qPCR SYBR Green Master Mix (Vazyme). Relative gene expression was quantified using the $2^{-\Delta\Delta Ct}$ method. *OsActin1* served as an internal control for expression normalization. Primers used are listed in Supplemental Table 2.

5′ RACE assay

5′ RACE was modified according to our method for low-input degradome library construction. In brief, polyadenylated RNAs were purified from total RNA, and those with a 5′ monophosphate were ligated to an RNA adapter at their 5′ end using T4 RNA ligase 1 (NEB). After ligation, first-strand cDNA was reverse transcribed using the oligo-dT_{25}VN primer and then amplified through seven cycles of PCR with the RACE F1 and R1 primers. To confirm the site of mRNA cleavage, nested PCR was performed using the reverse gene-specific primer (RACE R2 primer) and the 5′ adaptor primer (RACE F2 primer). Nested PCR primers for 5′ RACE of *OsARF7-OE* lines were the RACE F2 primer and RACE R3 primer, which can distinguish *OsARF7 transcripts derived from the* overexpression vector from endogenous *OsARF7* transcripts. The PCR products were purified and cloned into a T vector with the TA/Blunt-Zero Cloning Kit (Vazyme), and the recombinant clones were Sanger sequenced with the M13F primer. RNA adaptor and primer sequences are listed in Supplemental Table 2.

Pollen fertility analysis and paraffin sectioning

For pollen staining, anthers from stages 10–12 were crushed in an iodine–potassium iodide solution containing 1% (w/v) I_2 and 8% (w/v) KI. The stained pollen grains were collected with a pipette and transferred to a new slide. Images were captured with a microscope.

Panicles and spikelets at different developmental stages were chosen as described previously (Zhang et al., 2011a; Jiang et al., 2020). Spikelets were immediately fixed in FAA solution (3.7% formaldehyde, 5% acetic acid, and 50% ethanol) at 4°C overnight and then automatically dehydrated in a LEICA ASP200S tissue processor, embedded in paraffin (Sigma-Aldrich), and sectioned to 10-μm thickness using a LEICA RM2255 rotary microtome. After dewaxing, rehydration, and staining with eosin, the sections were observed under a microscope.

Northern blotting of osphasiR1_1

Northern blotting was performed as described previously (Mi et al., 2008). In brief, RNAs with high molecular weight were removed from the total RNA using PEG8000. The purified RNAs were treated with DNase I (Vazyme) for 20 min and precipitated with the help of glycogen. The resolved RNAs were loaded on 15% denaturing PAGE gels. After electrophoresis, RNAs were transferred onto Hybond-N+ membranes (GE Healthcare, USA) by UV treatment. Probes were ^{32}P end-labeled with T4 PNK, and hybridization signals were detected using the Personal Molecular Imager system (Bio-Rad). The sequences of the osphasiR1_1 and U6 RNA probes are listed in Supplemental Table 2.

RNA *in situ* hybridization

RNA *in situ* hybridization was performed as described previously, with minor modifications (Wu et al., 2020). Spikelets (2 mm in length) were fixed in FAA solution (30% ethanol, 1.85% formaldehyde, and 5% acetic acid) overnight at 4°C, dehydrated in a graded ethanol series, and embedded in Paraplast Plus (Merck). Probes for the gene regions were synthesized using a Digoxigenin Labeling Kit (Roche, USA) according to the manufacturer's instructions. Primer sets for the probes and phasiRNAs that were modified with locked nucleic acid (LNA) and digoxigenin (DIG) at the 3′ and 5′ termini are listed in Supplemental Table 2. Tissue cross-sections (10-μm thickness) were prepared using a rotary microtome. A portion of the sections was stained with 0.1% (w/w) safranin to identify the anthers. Sections mounted on slides were subjected to gradient dewaxing with xylene. Slides were incubated with 0.5 μg/ml proteinase K (Sigma) for 15 min at 37°C and then acetylated with 1.5% (v/v) triethanolamine in a solution of 0.25% (v/v) concentrated hydrochloric acid and 0.25% (v/v) acetic anhydride. Gene probes (0.8 μg/ml) or 25 nM

(F) Volcano plot of differentially expressed genes (DEGs) (*OsARF7^{mu}-OE*#3 vs. WT) in M2 anthers ($|\log_2 FC| \geq 1$, FDR < 0.05). Red and blue dots represent upregulated and downregulated genes, respectively.

(G and H) RT–qPCR validation of the expression of *$Ub_{L40}4$* and *cHSP70-4* (n = 3 biological replicates; *t*-test, $^{**}P < 0.01$).

(I) Schematic of *$Ub_{L40}4$* and *cHSP70-4* promoters (1.45-kb region upstream of the start codon) with predicted auxin response elements (*AuxRE*s). Truncated promoter fragments (−1450 to −700 and −750 to −1 bp) are indicated.

(J) Schematic of the dual-luciferase reporter system for the promoter activation assay.

(K and L) Relative LUC activity in rice protoplasts co-transformed with effector and reporter vectors. Only full-length promoters and truncated fragments containing *AuxRE*s were activated by OsARF7. n = 3 biological replicates; *t*-test, $^{**}P < 0.01$; ns indicates no significant difference.

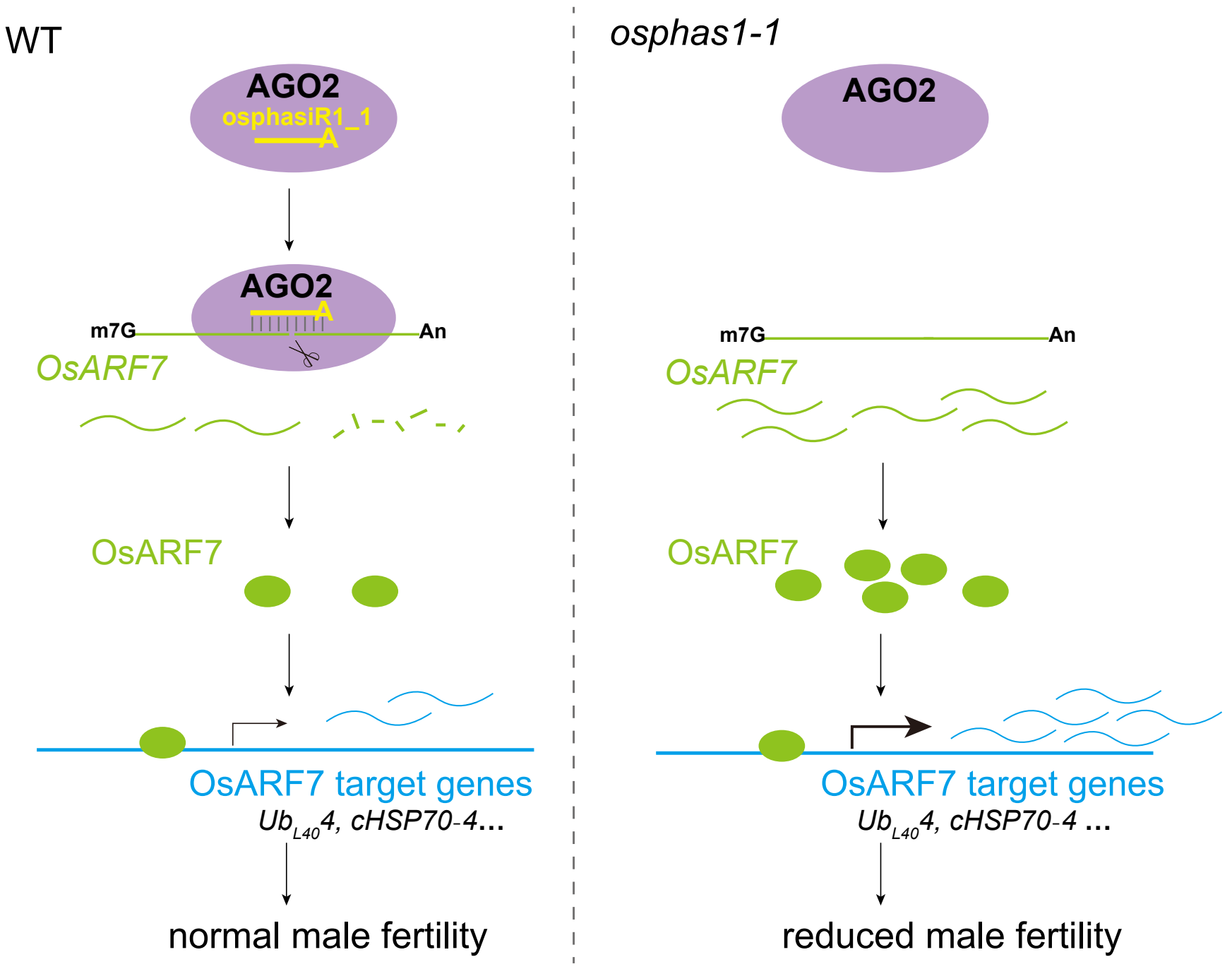

Figure 7. Model of male fertility regulation by osphasiR1_1 in rice.
In wild-type (WT) rice, osphasiR1_1 (5′-A phasiRNA) is loaded into OsAGO2, which directly cleaves *OsARF7* transcripts. This cleavage restricts *OsARF7* expression to an appropriate level, preventing hyperactivation of target genes (e.g., *Ub$_{L40}$4* and *cHSP70-4*) and ensuring normal male fertility. When osphasiR1_1 is knocked out or OsAGO2 is defective, *OsARF7* transcripts are not cleaved and accumulate to abnormally high levels. The overexpressed OsARF7 hyperactivates downstream fertility-related target genes, leading to defective male germ cell development and reduced male fertility.

phasiRNA probes were used for hybridization overnight at 55°C. The slides were washed twice with 50% (v/v) formamide in 2× saline–sodium citrate (SSC) for 30 min at 55°C and incubated with a buffer containing 10 μg/ml RNase A for 30 min at 37°C to remove unhybridized RNA probes. Next, the slides were washed twice with 2× SSC and 0.2× SSC for 30 min at 55°C. The blocking steps and detection of hybridized transcripts, which required anti-digoxigenin antisera conjugated to alkaline phosphatase (Roche Anti-Digoxigenin-AP, NBT/BCIP), were performed according to the manufacturer's protocol. The signal was detected after 3–16 h of incubation at 30°C.

Transcriptional activation assay in yeast

The full-length coding sequence of *OsARF7*, as well as its N-terminal and C-terminal fragments, was cloned into the yeast expression vector *pGBKT7* to generate *pGBKT7-OsARF7* (full length), *pGBKT7-OsARF7N* (N-terminal fragment), and *pGBKT7-OsARF7C* (C-terminal fragment). The empty *pGBKT7* vector served as a negative control. The recombinant plasmids were transformed into the yeast strain AH109 using the lithium acetate method. Positive transformants were selected on SD/−Trp dropout medium. To assess transcriptional activation, yeast colonies grown on SD/−Trp plates were serially diluted to concentrations of 10^0, 10^{-1}, 10^{-2}, and 10^{-3} and then spotted onto SD/−Trp (transformation control) and SD/−Trp−His−Ade media. Plates were incubated at 30°C for 3–5 days before observation.

Dual-luciferase reporter assay

The dual-luciferase assay was performed as described previously, with minor adjustments (Fang et al., 2025). For transcriptional activation analysis, the full-length *OsARF7* cDNA was cloned into the *pCAMBIA1300-35S-BD* (*GAL4*) vector to generate the effector construct. The reporter vector *pGreenII-0800-4×GAL4-mini35Spro-LUC-35Spro-REN* contained 4 copies of the GAL4 binding motif. The empty *pCAMBIA1300-35S-BD* vector served as a negative control. For assays of target genes, *OsARF7* was cloned into the *pCAMBIA1300-35S* vector to generate the effector construct. Promoter fragments of *cHSP70-4* and *Ub$_{L40}$4* (regions −1450 to −1, −1450 to −700, and −750 to −1 bp relative to the ATG start codon) were amplified and cloned into the *pGreenII 0800-LUC* vector. The empty *pCAMBIA1300-35S* vector was used as a negative control. The transfected *Nicotiana benthamiana* leaves were sprayed with luciferin solution, and after 2 min of reaction, fluorescence was detected using a Zeiss LSM 800 confocal microscope. LUC and *Renilla* luciferase activities were measured using a Dual-LUC Assay Kit (Yeasen, 11402ES80) according to the manufacturer's instructions. Transcriptional activity was quantified by calculating the LUC/*Renilla luciferase* ratio. Three independent biological replicates were performed for each sample. All primer sequences used are listed in Supplemental Table 2.

Observations of chromosomes at meiosis

Meiotic chromosomes were observed as described previously (Li et al., 2025). Young panicles of ZH11 and *OsARF7^{mu}-OE* plants were fixed in Carnoy's solution (ethanol:glacial acetic acid, 3:1, v/v). Meiotic anthers were crushed with forceps in 45% acetocarmine solution on glass slides and then covered with a coverslip. After staging with a light microscope, the slides were frozen in liquid nitrogen, and the coverslip was removed rapidly with a razor. Chromosomes were stained with 4′,6-diamidino-2-phenylindole, and images were captured with an Olympus BX61 epifluorescence microscope equipped with a CCD camera.

mRNA sequencing

RNA-seq libraries were constructed as described previously (Jiang et al., 2020). In brief, total RNA was used for reverse transcription and cDNA second-strand synthesis using SuperScript II reverse transcriptase (Invitrogen, 18064-14) and template-switching oligos. After PCR preamplification and purification with AMPure XP beads (Beckman, A63880), the products were subjected to fragmentation and adaptor ligation using the TruePrep DNA Library Prep Kit (Vazyme, TD503). PCR amplification was then performed with index-containing primers, and 300- to 800-bp products were purified with AMPure XP beads. Libraries were sequenced in paired-end mode on an Illumina NovaSeq platform at Annoroad Gene Technology (Beijing, China).

Analysis of mRNA sequencing data

Data were analyzed as described previously, with minor modifications (Jiang et al., 2020). Low-quality reads were removed, and adapters were trimmed using Trimmomatic (v0.36). Clean RNA reads were then mapped to the reference genome (MSU v7) with default parameters using TopHat v2.1.1. The expression level of each gene was normalized to fragments per kilobase of transcript per million mapped reads. Differentially expressed genes were identified using Cuffdiff (fold change $\geq$ 2.0 and false discovery rate $\leq$ 0.05).

DATA AND CODE AVAILABILITY

Raw mRNA sequencing data have been deposited in two public databases: the NCBI Sequence Read Archive (PRJNA1404157; https://www.ncbi.nlm.nih.gov/sra/PRJNA1404157) and the China National Genomics Data Center (CRA039169; https://ngdc.cncb.ac.cn/gsa/browse/CRA039169).

FUNDING

This work was supported by the Natural Science Research Project of Universities in Anhui Province (2022AH030094), the National Natural Science Foundation of China (32200283), and the Anhui Agricultural University Startup Fund (rc422202).

ACKNOWLEDGMENTS

We thank Professor Beijiu Cheng from Anhui Agricultural University and Professor Zhong Zhao from the University of Science and Technology of China for their valuable suggestions on the experimental design. No conflict of interest declared.

AUTHOR CONTRIBUTIONS

P.J. and L.S. designed this study. L.S., K.Q., R.X., and J.Z. performed the majority of the experiments, and the other authors contributed to the paddy field work, rice transformation, and assistance with experiments; Y.L. helped with the RNA *in situ* hybridization and chromosome observation; P.W., Y.Q., and H.J. provided strong support and guidance for this study; and P.J. wrote the manuscript.

SUPPLEMENTAL INFORMATION

Supplemental information is available at *Plant Communications Online*.

Received: October 8, 2025
Revised: November 10, 2025
Accepted: February 28, 2026
Published: March 3, 2026

REFERENCES

Allen, E., Xie, Z., Gustafson, A.M., and Carrington, J.C. (2005). microRNA-Directed Phasing during Trans-Acting siRNA Biogenesis in Plants. Cell **121**:207–221.

Araki, S., Le, N.T., Koizumi, K., Villar-Briones, A., Nonomura, K.I., Endo, M., Inoue, H., Saze, H., and Komiya, R. (2020). miR2118-dependent U-rich phasiRNA production in rice anther wall development. Nat. Commun. **11**:3115.

Attia, K.A., Abdelkhalik, A.F., Ammar, M.H., Wei, C., Yang, J., Lightfoot, D.A., El-Sayed, W.M., and El-Shemy, H.A. (2009). Antisense Phenotypes Reveal a Functional Expression of OsARF1, an Auxin Response Factor, in Transgenic Rice. Curr. Issues Mol. Biol. **11**:I29–I34.

Bélanger, S., Martín, A.C., Marchant, D.B., Zhan, J., McGregor, M., Smedley, M., Hayta, S., Moore, G., and Meyers, B.C. (2025). DICER-LIKE 5 loss causes thermosensitive male sterility in durum wheat and reveals an AU-rich motif guiding 24-nt phasiRNA biogenesis. Proc. Natl. Acad. Sci. USA **122**:e2504349122.

Boer, D.R., Freire-Rios, A., van den Berg, W.A.M., Saaki, T., Manfield, I.W., Kepinski, S., López-Vidrieo, I., Franco-Zorrilla, J.M., de Vries, S.C., Solano, R., et al. (2014). Structural basis for DNA binding specificity by the auxin-dependent ARF transcription factors. Cell **156**:577–589.

Borges, F., and Martienssen, R.A. (2015). The expanding world of small RNAs in plants. Nat. Rev. Mol. Cell Biol. **16**:727–741.

Chang, Z., Chen, Z., Yan, W., Xie, G., Lu, J., Wang, N., Lu, Q., Yao, N., Yang, G., Xia, J., and Tang, X. (2016). An ABC transporter, OsABCG26, is required for anther cuticle and pollen exine formation and pollen-pistil interactions in rice. Plant Sci. **253**:21–30.

Chen, L.L., and Kim, V.N. (2024). Small and long non-coding RNAs: Past, present, and future. Cell **187**:6451–6485.

Fang, X., and Qi, Y. (2016). RNAi in Plants: An Argonaute-Centered View. Plant Cell **28**:272–285.

Fang, Y., Liao, H., Wei, Y., Yin, J., Cha, J., Liu, X., Chen, X., Chen, L., Ma, Z., Zhang, J., et al. (2025). OsCDPK24 and OsCDPK28 phosphorylate heat shock factor OsHSFA4d to orchestrate abiotic and biotic stress responses in rice. Nat. Commun. **16**:6485.

Guilfoyle, T.J., and Hagen, G. (2007). Auxin response factors. Curr. Opin. Plant Biol. **10**:453–460.

Inukai, Y., Sakamoto, T., Ueguchi-Tanaka, M., Shibata, Y., Gomi, K., Umemura, I., Hasegawa, Y., Ashikari, M., Kitano, H., and Matsuoka, M. (2005). Crown rootless1, which is essential for crown root formation in rice, is a target of an AUXIN RESPONSE FACTOR in auxin signaling. Plant Cell **17**:1387–1396.

Jiang, P., Lian, B., Liu, C., Fu, Z., Shen, Y., Cheng, Z., and Qi, Y. (2020). 21-nt phasiRNAs direct target mRNA cleavage in rice male germ cells. Nat. Commun. **11**:5191.

Jung, K.H., Han, M.J., Lee, Y.S., Kim, Y.W., Hwang, I., Kim, M.J., Kim, Y.K., Nahm, B.H., and An, G. (2005). Rice Undeveloped Tapetum1 is a major regulator of early tapetum development. Plant Cell **17**:2705–2722.

Komiya, R., Ohyanagi, H., Niihama, M., Watanabe, T., Nakano, M., Kurata, N., and Nonomura, K.I. (2014). Rice germline-specific Argonaute MEL1 protein binds to phasiRNAs generated from more than 700 lincRNAs. Plant J. **78**:385–397.

Li, Y., Yang, J., Zhang, J., Xin, Z., Xu, R., Qiu, K., Wang, F., Sun, F., Jiang, H., Jin, W., et al. (2025). Heat shock protein 101 safeguards meiotic thermotolerance of male germlines in rice and Arabidopsis. Crop J.

Li, Z., Rao, M.J., Li, J., Wang, Y., Chen, P., Yu, H., Ma, C., and Wang, L. (2022). CRISPR/Cas9 Mutant Rice Ospmei12 Involved in Growth, Cell Wall Development, and Response to Phytohormone and Heavy Metal Stress. Int. J. Mol. Sci. **23**:16082.

Lian, B., Huang, T., Huang, W., Jiang, P., Jin, W., and Qi, Y. (2025). Spatiotemporal regulation of target mRNA cleavage by 21-nt phasiRNAs in maize anthers. Proc. Natl. Acad. Sci. USA **122**:e2422647122.

Liu, Y., Teng, C., Xia, R., and Meyers, B.C. (2020). PhasiRNAs in Plants: Their Biogenesis, Genic Sources, and Roles in Stress Responses, Development, and Reproduction. Plant Cell **32**:3059–3080.

Mao, X., Yu, H., Xue, J., Zhang, L., Zhu, Q., Lv, S., Feng, Y., Jiang, L., Zhang, J., Sun, B., et al. (2025). OsRHS Negatively Regulates Rice Heat Tolerance at the Flowering Stage by Interacting with the HSP Protein cHSP70-4. Plant Cell Environ. **48**:350–364.

Mi, S., Cai, T., Hu, Y., Chen, Y., Hodges, E., Ni, F., Wu, L., Li, S., Zhou, H., Long, C., et al. (2008). Sorting of Small RNAs into Arabidopsis Argonaute Complexes Is Directed by the 5′ Terminal Nucleotide. Cell **133**:116–127.

Nonomura, K.I., Morohoshi, A., Nakano, M., Eiguchi, M., Miyao, A., Hirochika, H., and Kurata, N. (2007). A germ cell-specific gene of the ARGONAUTE family is essential for the progression of premeiotic mitosis and meiosis during sporogenesis in rice. Plant Cell **19**:2583–2594.

Panwalkar, P., Khire, A., and Shirsat, N. (2022). Quantification of microRNAs in Cells and Tissues Using Stem-Loop RT PCR and qPCR. Methods Mol. Biol. **2423**:51–58.

Sakamoto, T., Morinaka, Y., Inukai, Y., Kitano, H., and Fujioka, S. (2013). Auxin signal transcription factor regulates expression of the brassinosteroid receptor gene in rice. Plant J. **73**:676–688.

Shi, C., Zhang, J., Wu, B., Jouni, R., Yu, C., Meyers, B.C., Liang, W., and Fei, Q. (2022). Temperature-sensitive male sterility in rice determined by the roles of AGO1d in reproductive phasiRNA biogenesis and function. New Phytol. **236**:1529–1544.

Song, X., Li, P., Zhai, J., Zhou, M., Ma, L., Liu, B., Jeong, D.H., Nakano, M., Cao, S., Liu, C., et al. (2012). Roles of DCL4 and DCL3b in rice phased small RNA biogenesis. Plant J. **69**:462–474.

Sun, L., Xiang, X., Yang, Z., Yu, P., Wen, X., Wang, H., Abbas, A., Muhammad Khan, R., Zhang, Y., Cheng, S., and Cao, L. (2018). OsGPAT3 Plays a Critical Role in Anther Wall Programmed Cell Death and Pollen Development in Rice. Int. J. Mol. Sci. **19**:4017.

Tamotsu, H., Koizumi, K., Briones, A.V., and Komiya, R. (2023). Spatial distribution of three ARGONAUTEs regulates the anther phasiRNA pathway. Nat. Commun. **14**:3333.

Teng, C., Zhang, H., Hammond, R., Huang, K., Meyers, B.C., and Walbot, V. (2020). Dicer-like 5 deficiency confers temperature-sensitive male sterility in maize. Nat. Commun. **11**:2912.

Toriba, T., Suzaki, T., Yamaguchi, T., Ohmori, Y., Tsukaya, H., and Hirano, H.-Y. (2010). Distinct Regulation of Adaxial-Abaxial Polarity in Anther Patterning in Rice. Plant Cell **22**:1452–1462.

Wang, B., Xue, J.S., Yu, Y.H., Liu, S.Q., Zhang, J.X., Yao, X.Z., Liu, Z.X., Xu, X.F., and Yang, Z.N. (2017). Fine regulation of ARF17 for anther development and pollen formation. BMC Plant Biol. **17**:243.

Wilmoth, J.C., Wang, S., Tiwari, S.B., Joshi, A.D., Hagen, G., Guilfoyle, T.J., Alonso, J.M., Ecker, J.R., and Reed, J.W. (2005). NPH4/ARF7 and ARF19 promote leaf expansion and auxin-induced lateral root formation. Plant J. **43**:118–130.

Wu, H., Qu, X., Dong, Z., Luo, L., Shao, C., Forner, J., Lohmann, J.U., Su, M., Xu, M., Liu, X., et al. (2020). WUSCHEL triggers innate antiviral immunity in plant stem cells. Science **370**:227–231.

Wu, M.F., Tian, Q., and Reed, J.W. (2006). Arabidopsis microRNA167 controls patterns of ARF6 and ARF8 expression, and regulates both female and male reproduction. Development **133**:4211–4218.

Yan, J., Gu, Y., Jia, X., Kang, W., Pan, S., Tang, X., Chen, X., and Tang, G. (2012). Effective Small RNA Destruction by the Expression of a Short Tandem Target Mimic in Arabidopsis. Plant Cell **24**:415–427.

Yu, Y., Wang, H., You, C., and Chen, X. (2026). Plant microRNA maturation and function. Nat. Rev. Mol. Cell Biol. **27**:55–70.

Yu, Y., Zhou, Y., Zhang, Y., and Chen, Y. (2018). Grass phasiRNAs and male fertility. Sci. China Life Sci. **61**:148–154.

Zhan, J. (2020). RDR6 Is Essential for Double-Strand Break Formation during Male Meiosis in Rice. Plant Cell **32**:3053–3054.

Zhan, J., and Meyers, B.C. (2023). Plant Small RNAs: Their Biogenesis, Regulatory Roles, and Functions. Annu. Rev. Plant Biol. **74**:21–51.

Zhang, D., Luo, X., and Zhu, L. (2011). Cytological analysis and genetic control of rice anther development. J Genet Genomics **38**:379–390.

Zhang, M., Ma, X., Wang, C., Li, Q., Meyers, B.C., Springer, N.M., and Walbot, V. (2021). CHH DNA methylation increases at 24-PHAS loci depend on 24-nt phased small interfering RNAs in maize meiotic anthers. New Phytol. **229**:2984–2997.

Zhang, X., Zhao, H., Gao, S., Wang, W.C., Katiyar-Agarwal, S., Huang, H.D., Raikhel, N., and Jin, H. (2011). Arabidopsis Argonaute 2 Regulates Innate Immunity via miRNA393*-Mediated Silencing of a Golgi-Localized SNARE Gene, MEMB12. Mol. Cell **42**:356–366.

Zhang, Y.C., Lei, M.Q., Zhou, Y.F., Yang, Y.W., Lian, J.P., Yu, Y., Feng, Y.Z., Zhou, K.R., He, R.R., He, H., et al. (2020). Reproductive phasiRNAs regulate reprogramming of gene expression and meiotic progression in rice. Nat. Commun. **11**:6031.

Zhao, Z., Wang, C., Yu, X., Tian, Y., Wang, W., Zhang, Y., Bai, W., Yang, N., Zhang, T., Zheng, H., et al. (2022). Auxin regulates source-sink carbohydrate partitioning and reproductive organ development in rice. Proc. Natl. Acad. Sci. USA **119**:e2121671119.

Zheng, S., Li, J., Ma, L., Wang, H., Zhou, H., Ni, E., Jiang, D., Liu, Z., and Zhuang, C. (2019). OsAGO2 controls ROS production and the initiation of tapetal PCD by epigenetically regulating OsHXK1 expression in rice anthers. Proc *Natl Acad Sci U S A*. Proc. Natl. Acad. Sci. USA **116**:7549–7558.

Zhou, H., Zhou, M., Yang, Y., Li, J., Zhu, L., Jiang, D., Dong, J., Liu, Q., Gu, L., Zhou, L., et al. (2014). RNase ZS1 processes Ub_{L40} mRNAs and controls thermosensitive genic male sterility in rice. Nat. Commun. **5**:4884.

Plant Communications, Volume *7*

Supplemental information

A phased small interfering RNA acts in an OsAGO2-dependent manner to mediate *OsARF7* cleavage and promote male fertility in rice

Liang Shao, Keli Qiu, Rui Xu, Jiankai Zhou, Junwen Yang, Jun Liu, Yunfei Yang, Lingling Li, Biao Xie, Yu Wang, Jiaojiao Li, Xiaoshuang Liu, Meng Yuan, Yachun Yang, Yunfei Li, Pengcheng Wei, Haiyang Jiang, Yijun Qi, and Pengfei Jiang

A phased small interfering RNA acts in an OsAGO2-dependent manner to mediate *OsARF7* cleavage and promote male fertility in rice

Liang Shao[1,6], Keli Qiu[1,6], Rui Xu[1,6], Jiankai Zhou[1,6], Junwen Yang[1], Jun Liu[1], Yunfei Yang[1], Lingling Li[1], Biao Xie[1], Yu Wang[1,3], Jiaojiao Li[4], Xiaoshuang Liu[5], Meng Yuan[2], Yachun Yang[3], Yunfei Li[1], Pengcheng Wei[1,5]*, Haiyang Jiang[1]*, Yijun Qi[2]*, Pengfei Jiang[1]*

1. National Engineering Laboratory of Crop Stress Resistance Breeding, School of Life Sciences, Anhui Agricultural University, Hefei 230036, China

2. Center for Plant Biology, School of Life Sciences, Tsinghua University, Beijing 100084, China.

3. Anhui Province Key Laboratory of Rice Germplasm Innovation and Molecular Improvement, Anhui Academy of Agricultural Sciences, Hefei 230036, China

4. CAS Center for Excellence in Molecular Plant Sciences, School of Life Sciences, University of Science and Technology of China, Huangshan Road 443, Hefei 230027, China

5. College of Agronomy, Anhui Agricultural University, Hefei 230036, China

6. These authors contribute equally to this work.

*Correspondence: Pengcheng Wei (weipengcheng@gmail.com), Haiyang Jiang (hyjiang@ahau.edu.cn), Yijun Qi (qiyijun@tsinghua.edu.cn), Pengfei Jiang (jiangpengfei@ahau.edu.cn)

Figure S1. Seed-setting rate and vegetative growth of *osphas1-1* and *osphasiR1_1-STTM* mutants

Figure S2. Phylogenetic analysis of *OsARFs* and sequence complementarity with osphasiR1_1

Figure S3. Seed setting rate of *OsARF7^{WT}-OE* and *OsARF7^{mu}-OE* lines

Figure S4. Loss of function of *OsARF7* results in normal male fertility

Figure S5. PCR-based genome sequencing of *osago2* and *osago5c* at Cas9 target region

Figure S6. The male fertility and *OsARF7* expression in *osago2* and *osago5c* mutants

Figure S7. Seed setting rate of *osago2-3* and *osago2-3 OsARF7-RNAi* lines

Figure S8. Heat map of the top 100 differentially expressed genes (DEGs)

Figure S9. Expression of fertility-related genes in WT and *OsARF7^{mu}*-OE lines

Figure S10. Expression of *OsARF7* during anther development in *osphas1-1* and *OsARF7^{mu}-OE*#3

Supplementary Table 1. Information of phasiRNAs which have targets degradome signal at top 30 in different rice varieties

Supplementary Table 2. Primers used in this study

Supplementary Table 3. Summary of the RNA-Seq data

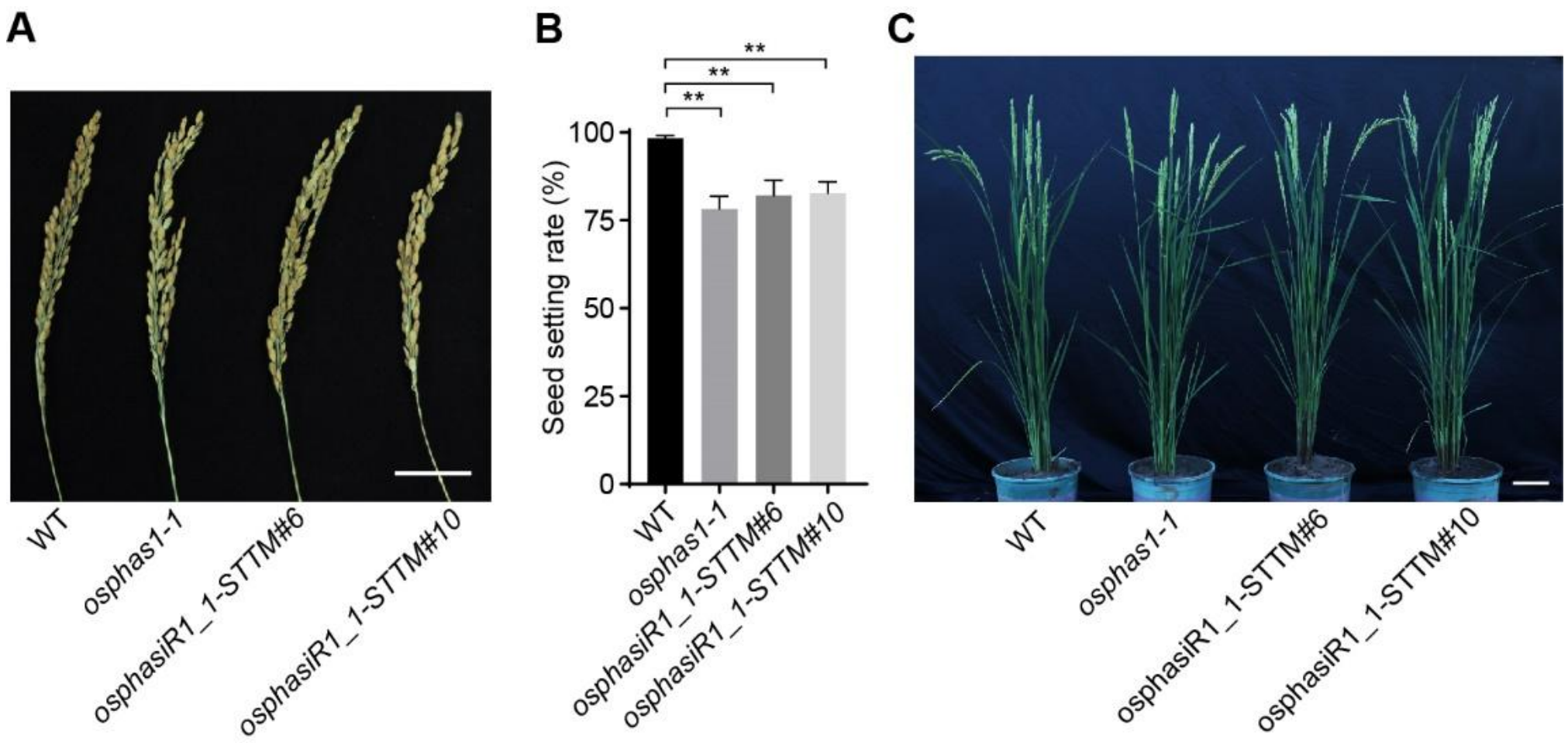

Figure S1. Seed-setting rate and vegetative growth of *osphas1-1* and *osphasiR1_1-STTM* mutants

(A-B) Panicles and Statistical analysis of seed-setting rate of WT and osphasiR1_1 mutants. Each two panicles from 5 seedlings of each mutant were used, t-test **P < 0.01. Bar = 4 cm.

(C) Representative images of 83-day-old WT, *osphas1-1*, and osphasiR1_1-STTM lines grown under long-day conditions (Hefei, China). Bar = 10 cm.

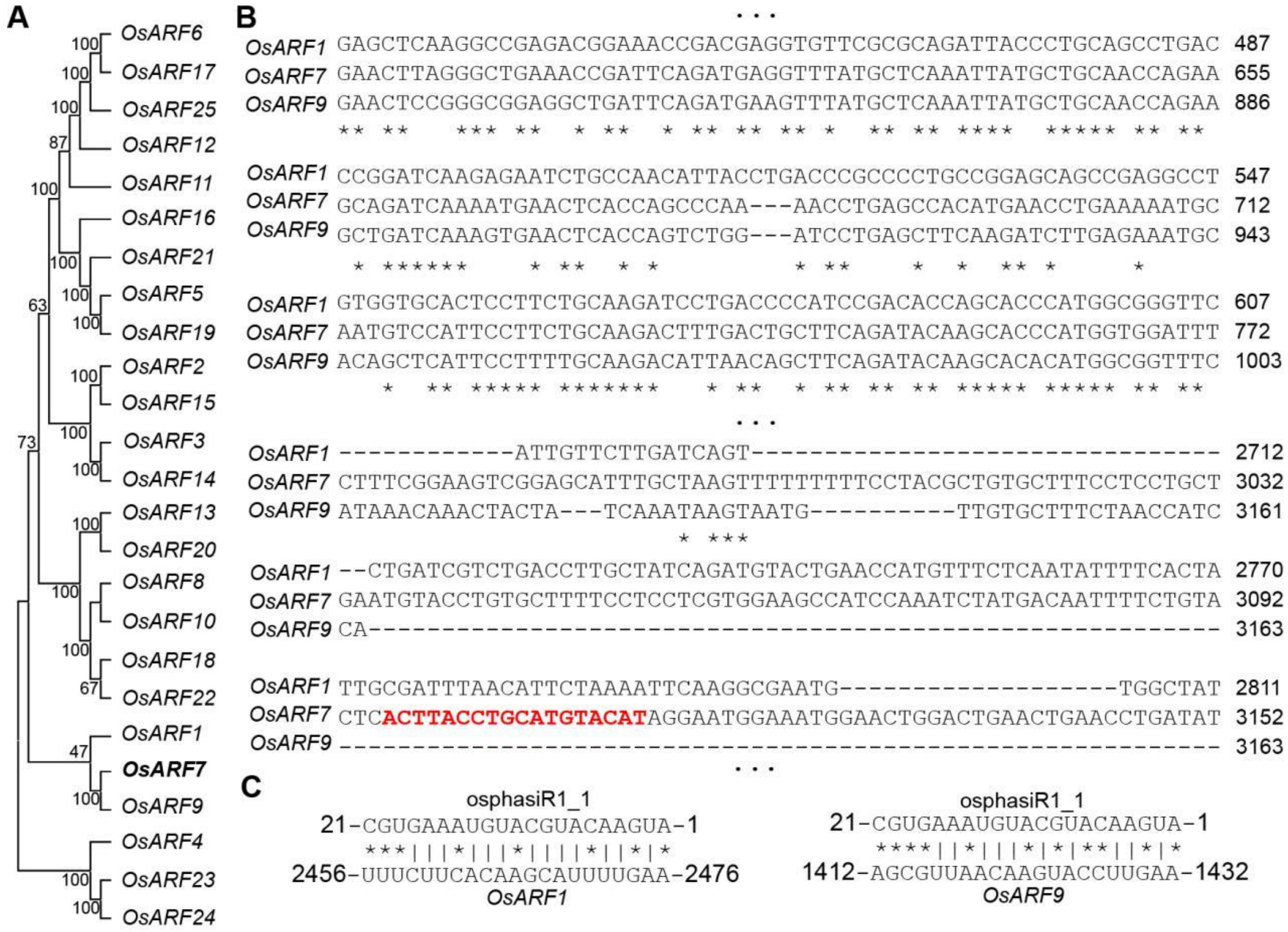

Figure S2. Phylogenetic analysis of *OsARFs* and sequence complementarity with osphasiR1_1

(A) Phylogenetic tree of *OsARFs* constructed using Neighbor-Joining (NJ) method.

(B) Sequence alignment of *OsARF1*, *OsARF7* and *OsARF9*. The sequences of osphasiR1_1 are shown in red.

(C) The mRNA sequences of *OsARF1* and *OsARF9* that exhibit maximum complementarity to osphasiR1_1.

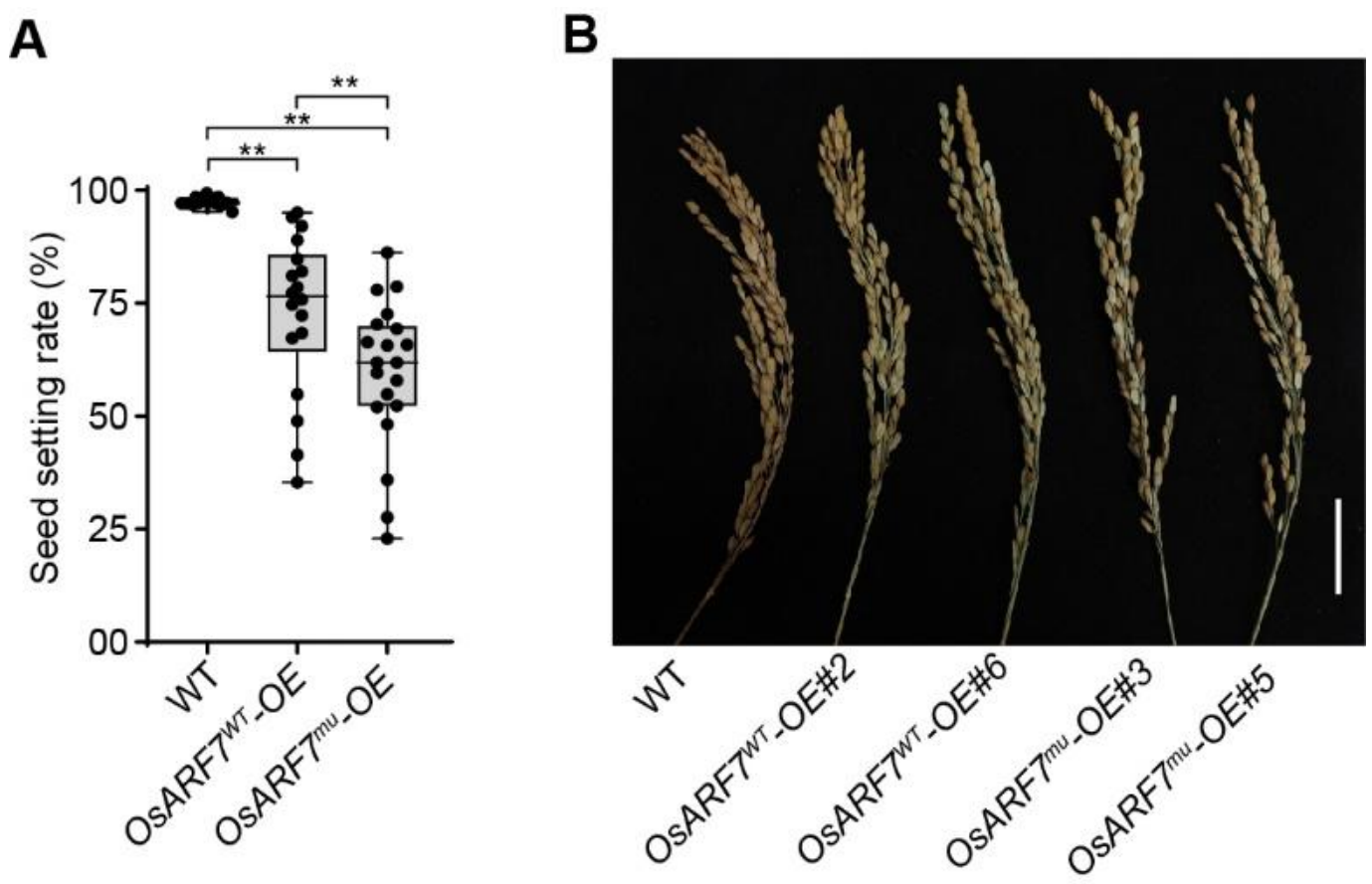

Figure S3. Seed setting rate of *OsARF7^{WT}-OE* and *OsARF7^{mu}-OE* lines

(A) Seed setting rate of WT, *OsARF7^{WT}-OE* and *OsARF7^{mu}-OE* lines at T_2 generation. At least 3 homozygous plants of each T_2 line were used, and each black solid dot indicates the average value of one line. T-test, $^{**}P < 0.01$.

(B) Panicles of WT as well as each two *OsARF7^{WT}-OE* and *OsARF7^{mu}-OE* lines after grain filling. Bar = 4 cm.

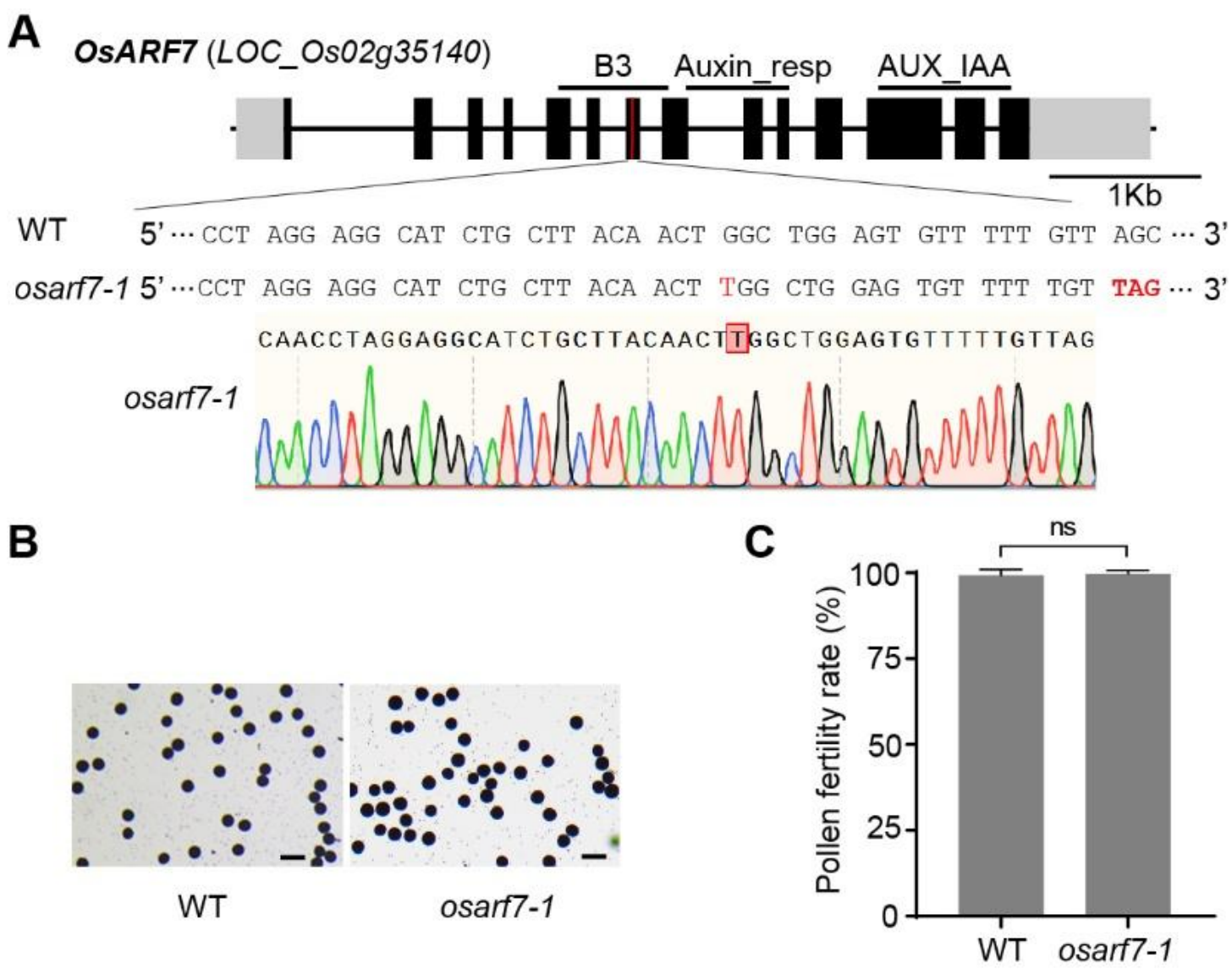

Figure S4. Loss of function of *OsARF7* results in normal male fertility

(A) Gene structure of *OsARF7*. The red vertical lines indicate the Cas9 target site of *OsARF7*.

(B-C) I_2-KI staining and statistics of pollen fertility of WT and *osarf7-1* mutant. Ten plants and 10 anthers per plant were used. T-test, ns indicates no significant difference. Bar = 200 μm

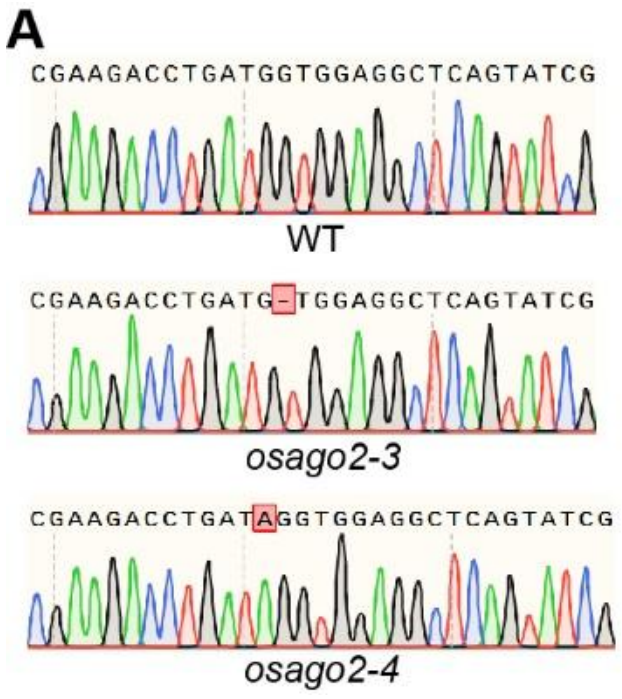

Figure S5. PCR-based genome sequencing of *osago2* and *osago5c* at Cas9 target region

Black lines highlighted in red background indicate deleted bases in the mutants, and nucleotide highlighted in red background indicates inserted base in the mutants.

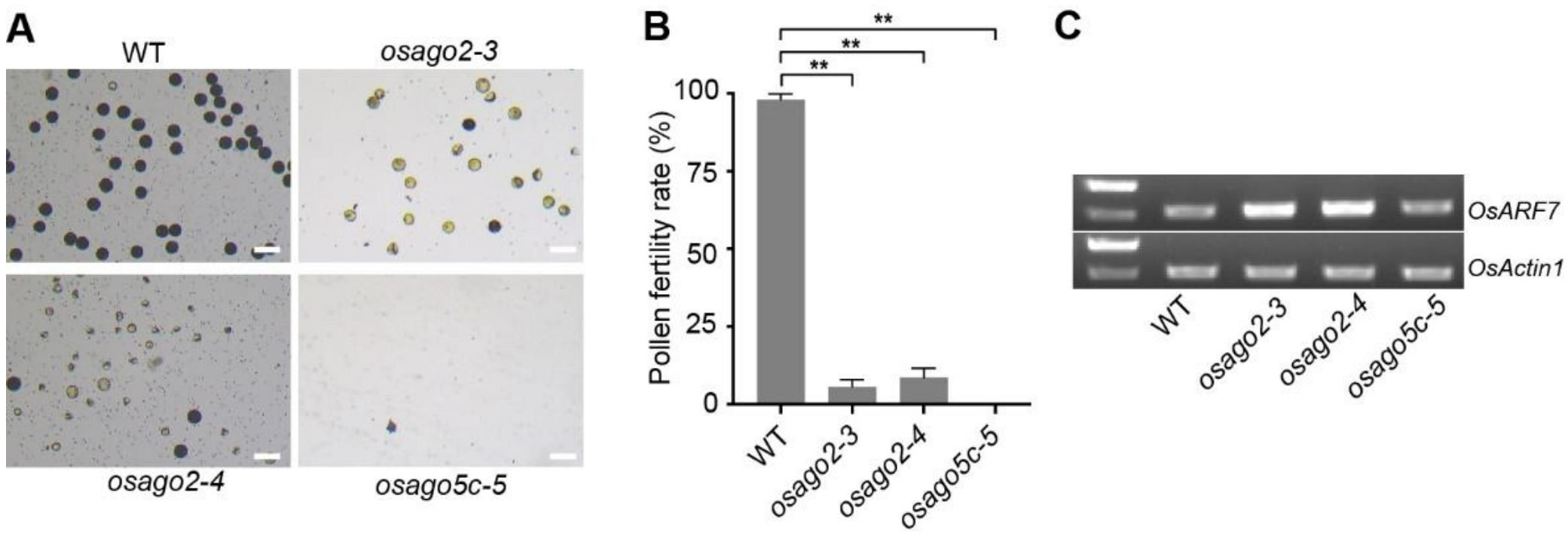

Figure S6. The male fertility and *OsARF7* expression in *osago2* and *osago5c* mutants

(A-B) I_2-KI staining and statistics of pollen fertility of WT, *osago2* and *osago5c* mutants. Ten plants and 10 anthers per plant were used. T-test, **P < 0.01. Bar = 200 µm.

(C) Semi-quantitative PCR analysis of *OsARF7* expression in *osago2* and *osago5c* mutants.

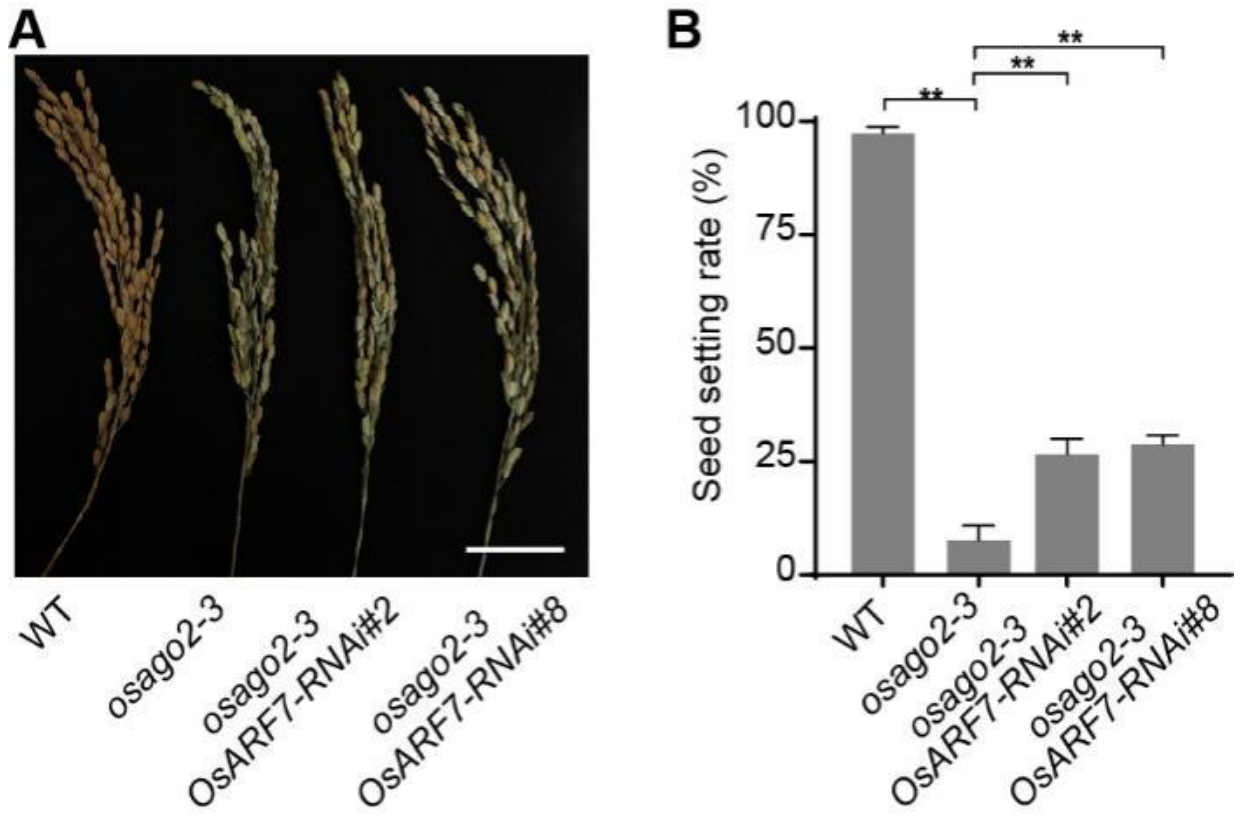

Figure S7. Seed setting rate of *osago2-3* and *osago2-3 OsARF7-RNAi* lines

(A) Panicles of WT, *osago2-3* and *osago2-3 OsARF7-RNAi* lines after grain filling. Bar = 4 cm.

(B) Seed setting rate. Ten plants and 3 panicles per plant were used. T-test, **P < 0.01.

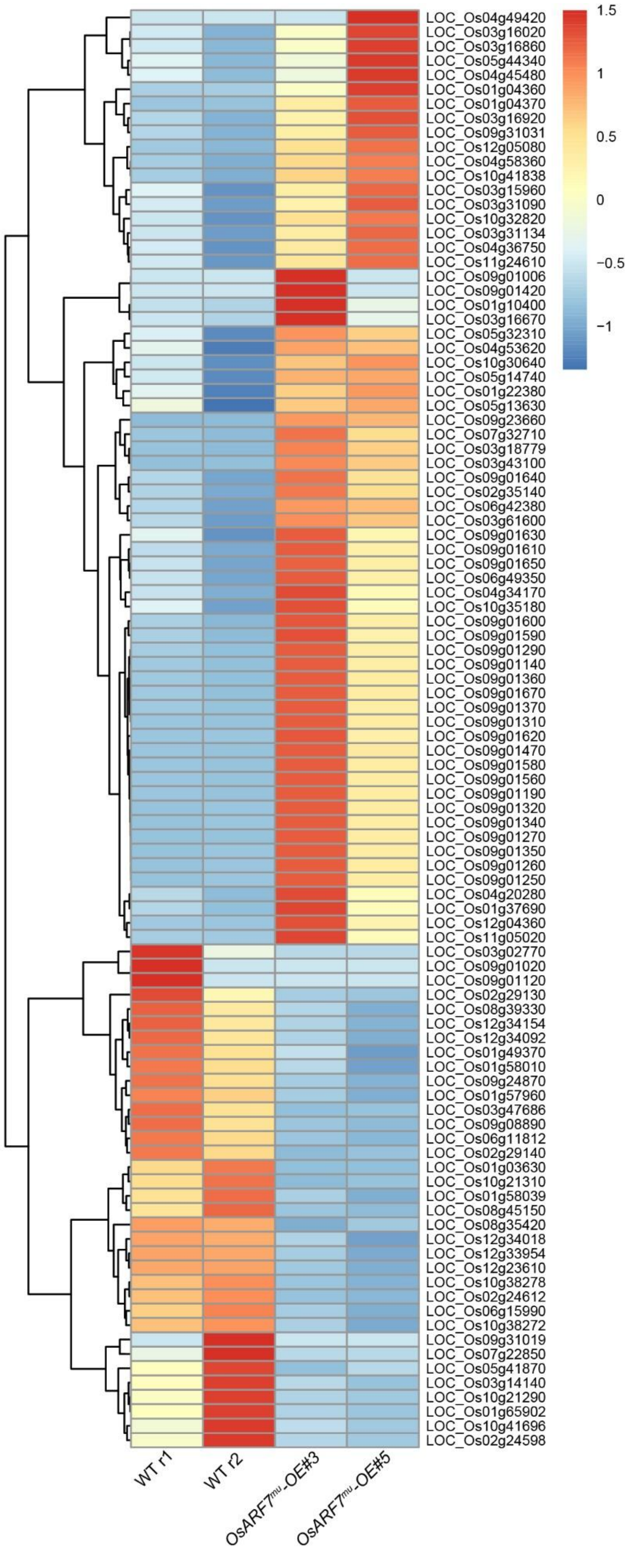

Figure S8. Heat map of the top 100 differentially expressed genes (DEGs)

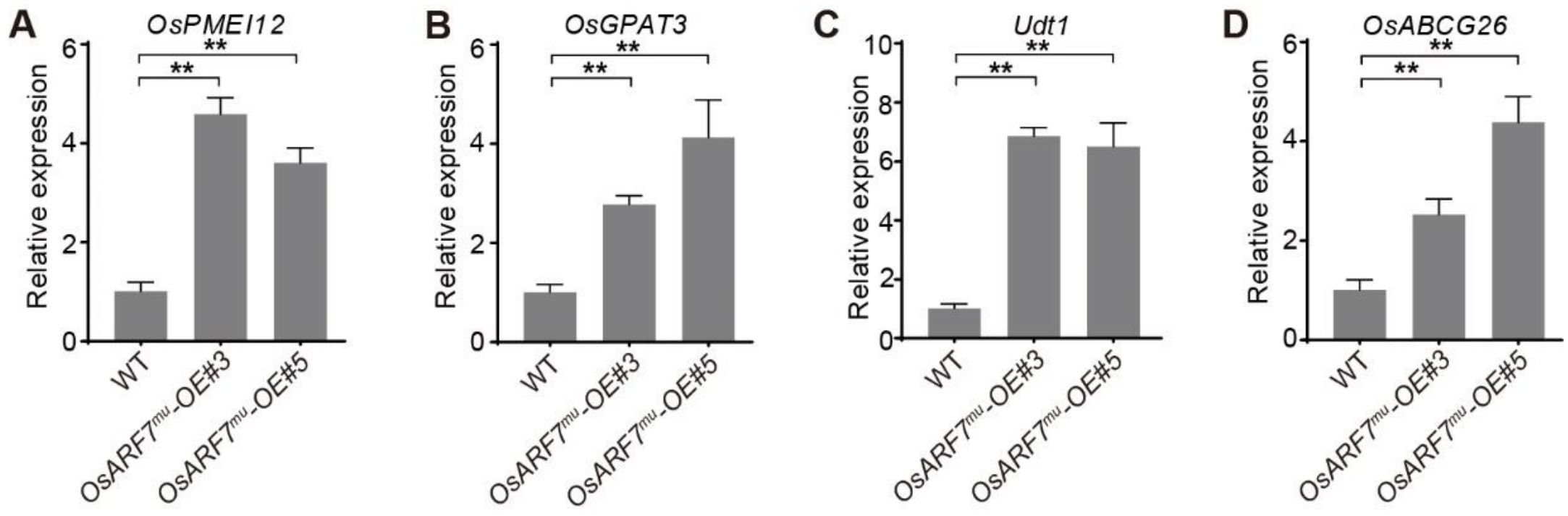

Figure S9. Expression of fertility-related genes in WT and *OsARF7^{mu}-OE* lines

N=3, biological replicates, t-test, $**P < 0.01$.

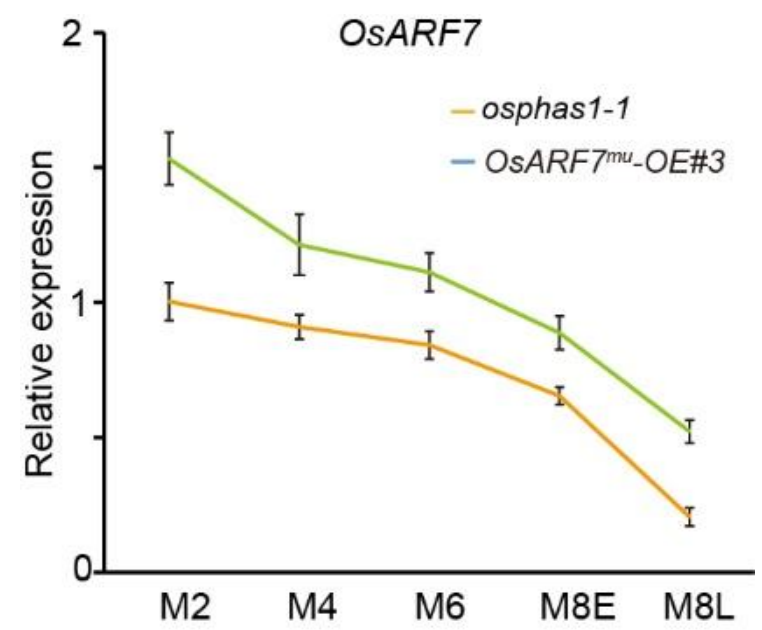

Figure S10. Expression of *OsARF7* during anther development in *osphas1-1* and *OsARF7^{mu}-OE*#3

N=3, biological replicates.

Supplementary Table 1. Information of phasiRNAs which have targets degradome signal at top 30 in different rice varieties

*phasiRNA*_ID	5' base	Target_ID	Cleavage site	Nipponbare		3037		HHZ		target description
				Total counts	Ranking	Total counts	Ranking	Total counts	Ranking	
21PHAS282_404(+)	*C*	LOC_Os12g33962	1031	6984	**1st**	3365	**1st**	2436	**1st**	tatC-like protein ymf16,
21PHAS672_488(-)	C	LOC_Os03g18820	1832	1390	**3rd**	2090	**2nd**	1787	**2nd**	glycosyltransferase
21PHAS277_639(+)	C	LOC_Os12g42550	1159	537	**9th**	1799	**3rd**	1053	**3rd**	methyl-CpG binding
21PHAS16_214(-)	C	LOC_Os02g02870	1193	732	**6th**	1197	**4th**	885	**4th**	glycine-rich protein 2
21PHAS511_132(-)	C	LOC_Os03g48930	776	1496	**2nd**	684	**5th**	673	**5th**	peptidase, T1 family
21PHAS1035_156(-)	*C*	LOC_Os06g44374	341	417	**13th**	283	**17th**	524	**6th**	SEC61 subunit gamma
21PHAS466_247(-) /osphasiR1_1	A	**LOC_Os02g35140**	**3104**	**486**	**10th**	**555**	**8th**	**480**	**7th**	**auxin response factor 7**
21PHAS46_349(-)	C	LOC_Os04g58890	1372	839	**5th**	581	**7th**	427	**8th**	expressed protein
21PHAS108_172(-)	G	LOC_Os10g02980	1574	262	**17th**	408	**10th**	379	**10th**	formin, putative
21PHAS1643_235(-)	C	LOC_Os05g39090	1994	290	**15th**	460	**9th**	340	**11th**	CAMK_CAMK_like.29
21PHAS1826_79(-)	C	LOC_Os02g47410	3529	691	**7th**	404	**11th**	211	**14th**	protein kinase
21PHAS944_81(+)	*C*	LOC_Os09g31970	1908	269	**16th**	290	**16th**	150	**19th**	HMGR putative

Supplementary Table 2. Primers used in this study

Gene	Primer	Sequence
Genotyping		
pUBI	F-primer	AATGCCAGCCTGTTAAACGCCGT
pUBI	R-primer	TGACCCGACAAACAAGTGCACG
osphas1-casdele	F-primer	GCATAACCTCATTGACCGAAAG
osphas1-casdele	R-primer	GTAGGACTATTCCCGACACCAC
ago2	F-primer	GCAACATTTGCTCTGTCATTTCC
ago2	R-primer	TTGACACGAAACAAACCATCTG
ago5c	F-primer	GCAGAAGCTGTTCGTGTCGGAGAC
ago5c	R-primer	GGAAAGAGGGTCAGGGGTGAGCT
osarf7	F-primer	GCAAGAGCTTGTGGCTAGAGATCTCC
osarf7	R-primer	GATGAATGCGTCACCGGCAACT
Vector construction		
OsARF7^{WT}-OE	F-primer	CAGCTATGACATGATTACGAATTCCAATGATAGCCAACCAC
OsARF7^{WT}-OE	R-primer	CTGCAGGTCGACTCTAGAGGATCCAGCAAACACGTTTGGAGGTCACG
OsARF7^{mu}-OE	R-primer	TCCTATCTACAAGGTGGTAAGTGAGTACAGAAAATTGTC
OsARF7^{mu}-OE	F-primer	TTACCACCTTGTAGATAGGAATGGAAATGGAACTGGAC
OsphasiR1_1-STTM	F-primer	CTCGGTACCGCACTTTACATCTAGCATGTTCATGTTGTTGTTGTTATGGTCTAGTTGTT
OsphasiR1_1-STTM	R-primer	TCACTGCAGATGAACATGCCTAATGTAAAGTGCATTCTTCTTCTTTAGACCATATTCTT
OsARF7-RNAi	F-primer	GTGCCAAGCTCCTGCAGGCTCGAGGTGGAAATCTTCAGCTGAGTCC
OsARF7-RNAi	R-primer	AGTAGTACGGTCCGTACCAGATCTACACCAGAAACAGCAGCCAGT
BD-ARF7	F-primer	ATATGGCCATGGAGGCCGAATTCATGGCTGGCTCCGTCGTTGCTGCT
BD-ARF7	R-primer	TTATGCGGCCGCTGCAGGTCGACTCAGCAATCCTTATCGGTGACGG
BD-ARF7-N	R-primer	TTATGCGGCCGCTGCAGGTCGACCTAGGACTCCAAGATGCATACTAT

BD-ARF7-C	F-primer	ATATGGCCATGGAGGCCGAATTCTGCATCTTGGAGTCCTAGCAAC
35S-BD-OsARF7	F-primer	CGGCAGGATCCACTAGTGAATTCATGGCTGGCTCCGTCGTTGCTGCT
35S-BD-OsARF7	R-primer	CCGGCCTCGAGGTCGACAAGCTTTCAGCAATCCTTATCGGTGACGG
35S-OsARF7	F-primer	GCCGCTCTAGAACTAGTGGATCCATGGCTGGCTCCGTCGTTGCT
35S-OsARF7	R-primer	AGGTCGACGGTATCGATAAGCTTTCAGCAATCCTTATCGGTGACG
pcHSP70-4	F1-primer	AGGTCGACGGTATCGATAAGCTTAAAACCGCCCTTCATACCACCGAG
pcHSP70-4	R1-primer	GCCGCTCTAGAACTAGTGGATCCCCCTGCCGTCCAAGTGGAGG
pcHSP70-4	F2-primer	AGGTCGACGGTATCGATAAGCTTCCTAAGTGCAAAATGCGCACACCT
pcHSP70-4	R2-primer	GCCGCTCTAGAACTAGTGGATCCCTTTCTTCTCTTCCGATCAAATC
pUb$_{L40}$*4*	F1-primer	AGGTCGACGGTATCGATAAGCTTATTACAGAGCAAAAAAGGAGT
pUb$_{L40}$*4*	R1-primer	GCCGCTCTAGAACTAGTGGATCCCGTTTGAAATGTAGATCTGAG
pUb$_{L40}$*4*	F2-primer	AGGTCGACGGTATCGATAAGCTTTACAAAATATAAAACACATT
pUb$_{L40}$*4*	R2-primer	GCCGCTCTAGAACTAGTGGATCCCTTGCCGCCGTCGAACGATCT
Stem-loop RT-qPCR		
OsphasiR1_1	RT-primer	GTCGTATCCAGTGCAGGGTCCGAGGTATTCGCACTGGATACGACGCACTT
OsphasiR1_1	F-primer	CGCCGGCATGAACATGCATGTA
OsphasiR1_1	R-primer	CCAGTGCAGGGTCCGAGGT
RT-qPCR		
OsARF7	F-primer	CGCATTCATCTTTCTGAGAGGTG
OsARF7	R-primer	ACTCAGACTGACTTGTTCTTGGT
OsAGO2	F-primer	TGGAGTTGGTGAATGCTGAC
OsAGO2	R-primer	CCACTGGCAGTTAGGCTGATC
cHSP70-4	F-primer	CTTTGATGCAAAGCGGCTGATCG
cHSP70-4	R-primer	ACTTCTTCTCCTCGCCCTTGTACG
OsABCG26	F-primer	TGGCCTCTGAAGCTTGTCAGTTC
OsABCG26	R-primer	GAGGAAATGATCCGATGGATTGCG

Ub$_{L40}$4	F-primer	TTCCCATCAGGTCTCACCACTG
Ub$_{L40}$4	R-primer	TTCTTCTTCAACCTCAGCTCCTTG
OsPMEI12	F-primer	CAAATTCTGCCGGTGCCTTTGC
OsPMEI12	R-primer	TGGCTTTCAATCGCTTGTGGAG
OsGPAT3	F-primer	GTTGGTTGATGTTGTGGGCTTCTC
OsGPAT3	R-primer	TGTCACCAGGTACACCTCCTTG
Udt1	F-primer	CTCTGCAGCTACAAAGTGCAAGTG
Udt1	R-primer	AACATCCTGCTCACCCTTCACCTC
OsActin1	F-primer	ACCCAAGAATGCTAAGCCAAGAG
OsActin1	R-primer	ACTTTGTCCACGCTAATGAAGAAAC
Semi-qRT-PCR		
OsARF7	F-primer	GCTCCAGAACTACCTCCGGTCT
OsARF7	R-primer	TCCTTGCATGATGACCTTGGT
OsAGO2	F-primer	TCGGGTACCGTTGAGCGCATTG
OsAGO2	R-primer	CTTGAAGAGCTCGTCCTTGACAGT
OsActin1	F-primer	AAGTACCCCATCGAGCATGGTATCG
OsActin1	R-primer	GAGCTGGTCTTGGCAGTCTCCATTT
5'RACE		
	5’ RNA adaptor	UUUCCCUACACGACGCUCUUCCGAUCUCCGAC
	RT-dT$_{25}$vn	CGAGCACAGAATTAATACGACT25VN
OsARF7	5'RACE-F1	TTCCCTACACGACGCTCTTC
OsARF7	5'RACE-R1	CGAGCACAGAATTAATACGACT
OsARF7	5'RACE-F2	TACACGACGCTCTTCCGATCT
OsARF7	5'RACE-R2	CACGCAGATTACCCCCATCACG
In stitu hybridization		
OsARF7	F-primer	TGTACAGGGAGCTATGGCATGCTTG
OsARF7	R-primer	GTAATACGACTCACTATAGGGCTGTAGCTTCCACAGCAGAGCTTATC
OsARF7	F- control	GTAATACGACTCACTATAGGGCTGTACAGGGAGCTATGGCATGCTTG
OsARF7	R-control	TGTAGCTTCCACAGCAGAGCTTATC
osphasiR1_1	ISH	5DiGN/GCACTTTACATGCATGTTCAT/3DiGN
osphasiR1_1	ISH-control	5DiGN/ ATGAACATGCATGTAAAGTGC/3DiGN

Supplementary Table 3. Summary of the RNA-Seq data

	**WT r1**	**WT r2**	***OsARF7^{mu}-OE*#3**	***OsARF7^{mu}-OE*#5**
Raw reads	45575108	48041304	22569594	52520112
Q20	97.75	97.59	98.12	98.07
Q30	92.42	91.51	94.39	94.25
Mapped reads	40672347(89.24%)	41311816(85.99%)	20585853(91.21%)	47322364(90.10%)
